# *Ziziphus jujuba* Mill. var. *spinosa* (Bunge) Hu ex H. F. Chou Seed Ameliorates Insomnia in Rats by Regulating Metabolomics and Intestinal Flora Composition

**DOI:** 10.3389/fphar.2021.653767

**Published:** 2021-06-16

**Authors:** Yue Hua, Sheng Guo, Hong Xie, Yue Zhu, Hui Yan, Wei-wei Tao, Er-xin Shang, Da-wei Qian, Jin-ao Duan

**Affiliations:** State Administration of Traditional Chinese Medicine Key Laboratory of Chinese Medicinal Resources Recycling Utilization, Jiangsu Collaborative Innovation Center of Chinese Medicinal Resources Industrialization, Nanjing University of Chinese Medicine, Nanjing, China

**Keywords:** insomnia, *Ziziphus jujuba* Mill. var. *spinosa* (Bunge) Hu ex H. F. Chou seed, metabolomics, intestinal flora, short-chain fatty acid

## Abstract

The seed of *Ziziphus jujuba* Mill. var. *spinosa* (Bunge) Hu ex H. F. Chou (ZSS) is often used as a traditional Chinese medicine for insomnia due to its sedative and hypnotic effects, but the mechanism underlying this effect has not been thoroughly elucidated. In this study, an insomnia model induced by intraperitoneal injection of DL-4-chlorophenylalanine suspension in Sprague-Dawley rats was adopted to investigate the therapeutic effect of ZSS extract. Metabolomics analyses of plasma and urine as well as 16S rRNA gene sequencing of the intestinal flora were performed. The relationships between the plasma and urine metabolites and the intestinal flora in insomnia rats were also analyzed. The results showed that changes in plasma and urine metabolites caused by insomnia were reversed after administration of ZSS, and these changes were mainly related to amino acid metabolism, especially phenylalanine metabolism. The results of 16S rRNA gene sequencing and short-chain fatty acid determination showed that the ZSS extract could reverse the imbalance of intestinal flora caused by insomnia and increase the contents of SCFAs in feces. All of these improvements are mainly related to the regulation of inflammation. Therefore, it is concluded that insomnia, which alters metabolic profiles and the intestinal flora, could be alleviated effectively by ZSS extract.

## Introduction

Insomnia is a ubiquitous and refractory sleep disorder characterized by frequent and persistent difficulty falling asleep and/or difficulty maintaining sleep, which leads to unsatisfactory sleep (Xu et al., 2019). The common symptoms are difficulty falling asleep, decreased sleep quality, reduced sleep time, and decreased memory and attention. With the accelerated pace of life and increased work pressure, the incidence of insomnia is increasing year by year. At present, approximately one-third of the world’s population suffers from insomnia of varying degrees, of which 4–6% meet the diagnostic criteria for insomnia (Chung et al., 2015). The prevalence of insomnia in China’s total population is lower than that in Western countries but similar to that in Asian countries, and the overall prevalence is approximately 15.0% (Cao et al., 2017). Insomnia is not only directly related to chronic pain, sleep apnea, cerebral infarction and adverse cardiometabolic risks (St-Onge et al., 2016) (obesity, hypertension, type 2 diabetes (Anothaisintawee et al., 2016), and coronary heart disease), but can also cause mental disorders such as anxiety and depression and even dependence on alcohol, nicotine and drugs, which seriously affect the quality of life of patients (Riemann et al., 2017). In summary, insomnia has become a prominent problem that threatens public health in China and the world, and it has increasingly attracted the attention of the government and the public (Araujo et al., 2017).

Commonly used drugs for insomnia are mainly divided into benzodiazepines (BZD) and nonbenzodiazepines (Qaseem et al., 2016). Currently, BZD are commonly used in the treatment of insomnia in Western medicine, but most of them have adverse reactions such as tolerance, addiction, and withdrawal symptoms. As a complementary and alternative therapy, traditional Chinese medicine (TCM) has the characteristics of fewer side effects and less dependence, and it has received increasing attention. Insomnia belongs to the category of “bu mei” in TCM and is related to the disharmony of yin and yang, ying-wei system disorders, and imbalance of viscera (Singh and Zhao, 2017). The most frequently used herb for insomnia is the seed of *Ziziphus jujuba* Mill. var. *spinosa* (Bunge) Hu ex H. F. Chou (ZSS, called Suan zao ren in China), and the most common herbal formulae include Suan zao ren tang and Wen dan tang, which are consistent with current clinical practice (Ni et al., 2019).

ZSS is a traditional Chinese herb used for both medicine and food and was first recorded in Shennong’s Classic of Materia Medica. It has been used for the treatment of insomnia for thousands of years in China. In line with herbal records, modern pharmacology has confirmed that ZSS has sedative and hypnotic (Jiang et al., 2007), antidepressant (Shi et al., 2019), anxiolytic (Li et al., 2019) and neuroprotective (Park et al., 2019) effects. Previous studies have shown that ZSS contains many flavonoids (Niu et al., 2010), saponins (Du et al., 2019a), alkaloids (Kang et al., 2016), and fatty oils (Xiao et al., 2018). Among them, flavonoids (such as spinosin) (Wang et al., 2010) and saponins (such as jujuboside A (Wang et al., 2015) and jujuboside B) were reported to have sedative and hypnotic effects and are believed to be the main active compounds for the treatment of insomnia in ZSS. However, these components are trace in ZSS, and their bioavailability is low, making it difficult to penetrate the blood-brain barrier. Therefore, the integration effect and the molecular mechanism of the interaction between ZSS and insomnia have not been clarified.

At present, the mechanism of insomnia treatment mainly focuses on regulating neurotransmitters (serotonin, norepinephrine, dopamine, glutamic acid, etc.) (Xu et al., 2018), affecting sleep-related factors (inflammation factors (Irwin et al., 2016), neurotrophic factors, etc.), adjusting hypothalamic-pituitary-adrenal axis hormone levels (corticotropin-releasing hormone; adrenocorticotropic hormone; cortisol), and improving the central nervous system structure (Morin, 2012; Levenson et al., 2015). However, there is also a “brain-gut-microbiota axis” in the human body, and the intestinal flora can affect the brain function of the host by regulating the abovementioned pathways (Cryan and Dinan, 2012; Osadchiy et al., 2019), which in turn affects mood, behavior and cognitive function (Martin and Mayer, 2017). It was confirmed that disorders of the intestinal flora can produce neurodegenerative diseases such as affective disorders, autism, Alzheimer’s disease, and Parkinson’s disease, as well as emotional disorders, such as cognitive and memory disorders (Mayer et al., 2015; Kundu et al., 2017). Current studies have shown that the intestinal flora is associated with insomnia, and short-term loss of sleep can have a subtle impact on the microbiota (Benedict et al., 2016). In addition, the microbiota and its metabolism are related to the host’s circadian rhythm, and they can also interact with each other (Li et al., 2018; Bishelisari and Keshavarziau, 2019). Sleep deprivation can cause disturbances in colon homeostasis (Gao et al., 2020), and the intestinal microbiota may program diurnal metabolic rhythms in the mouse small intestine through histone deacetylase 3 (Kuang et al., 2019). At the same time, metabolomics is an effective means to systematically analyze the changes in endogenous metabolites under exogenous stimulation, which can be applied to study the mechanism of disease and the intervention of drugs. This is consistent with the research methods of TCM, focusing on the investigation of holistic and multitarget mechanisms (More et al., 2015).

Changes to the microbial community in the gut have been proposed to promote metabolic disturbances that also occur after short periods of sleep loss. Therefore, we explored the mechanism of insomnia from the perspective of metabolomics and intestinal flora. In this study, ultra-performance liquid chromatography coupled with quadrupole time-of-flight mass spectrometry (UPLC-Q-TOF/MS) was used to investigate the plasma and urine metabolic profiles, 16S rRNA gene sequencing was used to investigate the composition of the intestinal flora, and gas chromatography-flame ionization detection (GC-FID) was used for the determination of short-chain fatty acids (SCFAs). Moreover, integrative analysis of the relationships between metabolites and specific intestinal flora profiles was performed to understand the potential regulatory mechanisms of oral ZSS extract to preliminarily explore the mechanism of ZSS in calming hearts and tranquilizing minds.

## Materials and Methods

### Materials

ZSS was collected from Hebei Province, China, in November 2018. The herb was identified as the seed of *Ziziphus jujuba* Mill. var. *spinosa* (Bunge) Hu ex H. F. Chou by Professor Jin-ao Duan, Nanjing University of Chinese Medicine. After collection, the ZSS voucher specimen (ZSS-20181122) was deposited in the Jiangsu Collaborative Innovation Center of Chinese Medicinal Resources Industrialization, Nanjing University of Chinese Medicine under closed and dry conditions at 25 ± 5°C.

### Preparation and Composition Determination of *Ziziphus jujuba* Mill. var. *spinosa* (Bunge) Hu ex H. F. Chou Seed Extract

The ZSS coarse powder was prepared, soaked in 2.5 times petroleum ether for 1 h, and then refluxed twice, each time for 2 h. The filtrate was merged, and the solvent was recovered to obtain the ZSS oil extract which contains 6.67 g of crude drug per ml. The filter residue was evaporated to dryness in the fume hood, and the off-white degreased residue was obtained. The residue was refluxed twice with 70% ethanol 8 times for 2 h each time. The ZSS ethanol solution was concentrated under reduced pressure to a concentration of approximately 4 g of crude drug per ml. The extracts were stored at − 20°C for further use, returned to room temperature and diluted with pure water to the required dose before use. Qualitative and quantitative analyses of the composition of the ZSS extract were performed by UPLC-Q-TOF/MS (Waters, United States) and HPLC-PDA-ELSD (Waters, United States; Alltech, United States), respectively. The apparatus characteristics and chromatographic conditions are provided in Supplementary Method S1.

### Animal Study

Healthy specific pathogen-free (SPF)-grade Sprague-Dawley rats (male, 180–220 g) were obtained from the Shanghai SLAC Laboratory Animal Co., Ltd, Shanghai, China (SCXK (Hu) 2017–0005). The animal experiment ethics committee of Nanjing University of Chinese Medicine has reviewed and approved the experiment with ethics application number 201905A029 which conformed to the Regulations on the Administration of Laboratory Animals issued by the State Science and Technology Commission and the Detailed Rules for the Implementation of the Administration of Medical Laboratory Animals issued by the Ministry of Health. All animals were kept under SPF conditions with a 12 h light/dark cycle and had free access to water and feed. The temperature and relative humidity of the environment were 23 ± 2°C and 60 ± 2%, respectively.

After one week of adaptive feeding, the rats were randomly divided into a control group (C, *n* = 6) and an insomnia model group (*n* = 42). Starting on the eighth day, the insomnia model group was intraperitoneally injected with DL-4-chlorophenylalanine suspension (PCPA, 350 mg/kg, Lot. C10438621, Macklin, Shanghai, China, prepared with 0.1% (w/v) Tween-80 normal saline) once a day for three consecutive days, while the same volume of normal saline was injected into the abdominal cavity of the control group.

The insomnia rats were randomly divided into seven groups (6 rats per group): the model group (M), positive group (POS, 1 mg/kg, diazepam, Lot. 14181201, Shanghai Shangyao Xinyi Pharmaceutical Factory Co., Ltd, Shanghai, China), and high-dose ZSS ethanol extract group (ZSSH, 20 g/kg), low-dose ZSS ethanol extract group (ZSSL, 5 g/kg), ZSS oil extract group (ZSSO, 5 g/kg, prepared with 0.5% (w/v) sodium carboxymethyl cellulose), high-dose ZSS ethanol + oil extract group (ZSSH + O), and low-dose ZSS ethanol + oil extract group (ZSSL + O). Among them, the dosage of ZSS extracts refers to the weight of the medicinal material. The dosage setting was based on the clinical use of Chinese medicine practitioners and literatures (Du et al., 2019b). The human equivalent dose (HED) of low dose is 0.81 g/kg, which is 3.2 times of the clinical dosage; while the HED of high dose is 3.23 g/kg, which is 12.9 times of the clinical dosage (Food and Drug Administration, 2005). From establishing the rat model, six animals were used in each group, and all groups were orally given the corresponding medicine at a dose of 10 ml/kg for 10 days. The rats in each treatment group were given ZSS extract or diazepam by oral administration, and the rats in the control group and the model group were given the same volume of normal saline. During the experiment, rat mental activity, hair changes, body weight, and intake of food and water were monitored. The concentration of 5-hydroxytryptamine (5-HT) after modeling and administration was determined to judge the success of the model and the effect of administration.

### Sample Collection

After fasting for 12 h on the last day of modeling, 500 μl of blood was collected from the rat orbits in the control group and the model group and placed in 1.5 ml centrifuge tubes. The blood was allowed to stand for 1 h and centrifuged at 1160 × *g* for 10 min, and the upper serum was taken and stored in a refrigerator at − 80°C for model verification. The 5-HT in the serum of the control group and model group after modeling was verified by using an enzyme-linked immunosorbent assay (ELISA) kit (Nanjing Jiancheng Bioengineering Institute, China).

On the evening of the sixth and seventh days of gavage, the rats were fasted in metabolic cages, and 12 h urine samples were collected and stored at − 80°C for urine metabolomics analysis. On the eighth and ninth days, each group received Morris water maze (MWM) acquisition trial and probe trial for behavioral index determination. After the last administration, fresh feces of each rat were collected one by one directly into the sterilization centrifuge tube to avoid the fresh feces from touching the hair and other pollutants. After collecting feces, the centrifuge tube was put into liquid nitrogen, and then transferred to − 80°C refrigerator for determination of intestinal flora and SCFAs, respectively.

After the experiment, the animals were euthanized using 2% pentobarbital sodium (prepared in normal saline, 40 mg/kg, intraperitoneal injection), and blood was collected from the abdominal aorta. After half of the blood was mixed uniformly in the ethylenediaminetetraacetic acid dipotassium salt anticoagulation tube, plasma was collected. The other half was added to the tube without anticoagulant and serum was taken. Both of plasma and serum were stored in a refrigerator at − 80°C. Among them, plasma was used for plasma metabolomics analysis, and serum was used for 5-HT determination with an ELISA kit.

### Behavioral Index Determination

According to the literatures (Suzuki et al., 2004; Vorhees and Williams, 2006), MWM acquisition and probe trials were performed with each group to obtain behavioral indexes. The rats were put into the water with their heads facing the pool wall, starting from the left or right sides of the target quadrant as the starting position. The acquisition training lasted for 60 s (s); if the rats could not find the platform within 60 s, the rats were guided to reach the platform and stayed on the platform for 10 s. Each rat was trained twice in the morning and afternoon. On the second day, the platform was removed, and the animals were randomly placed into the water from around the target quadrant. The ANY-maze animal behavior analysis system was used to record the movement track of animals in the water. The number of times the rat entered the platform area after the platform being removed, and the percentage of the distance and time to enter the target quadrant, were used as the detection indexes of spatial memory.

### Metabolomics Study on Plasma and Urine

After being thawed at room temperature, the plasma samples were extracted with three times the amount of acetonitrile to precipitate protein, while the urine samples were extracted with two times the amount of acetonitrile. The mixture was vortexed for 30 s and centrifuged at 15,000 × *g* for 15 min at 4°C. The supernatant was concentrated to dryness by vacuum centrifugation and then redissolved in 200 μl of 50% cold acetonitrile. The solution was vortexed, mixed and centrifuged again. The redissolved supernatant was transferred into liquid vials with intubation, and an aliquot of 2 μl was injected into UPLC-Q-TOF/MS for metabolism analysis. To monitor the stability of the instrument, two samples were selected randomly from each group and mixed in equal volume to obtain the quality control (QC) sample. Prior to sample injection, the QC sample was continuously injected six times to adjust and balance the system and was injected every six samples to monitor the stability of the analysis.

The separation was performed on an ACQUITY UPLC BEH C_18_ column (100 mm × 2.1 mm, 1.7 μm), which was maintained at 35°C, and the mobile phase was composed of 0.1% formic acid solution (A) and acetonitrile (B) at a flow rate of 0.4 ml/min. For plasma analysis, the gradient elution conditions were as follows: 0.0–3.0 min, 95–55% A; 3.0–13.0 min, 55–5% A; 13.0–14.0 min, 5% A; 14.0–14.1 min, 5–95% A; 14.1–15.0 min, 95% A. For urine analysis, the gradient elution conditions were as follows: 0.0–8.0 min, 95–70% A; 8.0–11.0 min, 70–30% A; 11.0–13.0 min, 30–5% A; 13.0–14.0 min, 5% A; 14.0–14.1 min, 5–95% A; 14.1–15.0 min, 95% A. Electrospray ionization (ESI) mass spectra were acquired in both positive and negative ionization modes. The mass spectra conditions were as follows: capillary voltage, 3.0 kV; cone voltage, 30 V; extraction voltage, 4.0 V; collision energy, 15–40 eV; desolvation temperature, 450°C; ion source temperature, 120°C; desolvation gas flow rate, 800 L/h; cone gas flow rate, 50 L/h; scan time, 0.3 s; interscan time, 0.02 s. High purity nitrogen and leucine-enkephalin (ESI+: 556.2771 m/z, ESI−: 554.2615 m/z) were the gas collision and locked mass solution, respectively.

The original chromatographic peak data obtained from mass spectrometry were processed using Masslynx V4.1 workstation, including noise removal, overlapping peak resolution, peak alignment, peak matching, standardization and normalization. The parameters were as follows: retention time (RT), 0.5–14 min; mass-to-charge ratio (m/z), 100–1000 Da; mass tolerance, 0.01 Da; intensity threshold, 50 counts; mass window, 0.05 Da; RT window, 0.2 min. The parameters of width at 5% height and peak-to-peak baseline noise were automatically calculated. The total peak area was normalized according the reference (Gardlo et al., 2016), that is, the ion intensities for each detected peak were normalized against the sum of the peak intensities within the sample. The data lists of RT, m/z and normalized peak area of each peak in positive and negative ion mode were generated, respectively.

The data were imported into EZinfo 2.0, and all variables were scaled to Pareto (par) for multivariate statistical analyses including principal component analysis (PCA), partial least squares-discriminant analysis (PLS-DA), and orthogonal partial least squares-discriminant analysis (OPLS-DA). The scatter points in S-plot of OPLS-DA and variables with VIP >1 were selected as potential biomarkers for further statistical analysis. T-test analysis was used to investigate whether the relative peak area of biomarkers had significant difference between the two groups, and the metabolites with statistical significance (*p* < 0.05) were selected. For the biomarkers that meet the above conditions, the databases, such as Human Metabolome Database (HMDB, http://www.hmdb.ca/), MetaboAnalyst database (http://www.metaboanalyst.ca/) and KEGG database (http://www.genome.jp/kegg/), were used to identify the potential markers and metabolic pathways.

### Intestinal Flora Analysis

According to the reference (Zhao et al., 2020), the intestinal flora diversity was sequenced and analyzed. The total microbial DNA was extracted from fresh fecal samples using the E. Z.N.A.^®^ Soil DNA Kit (D5625, Omega Biotek Inc, United States) according to the manufacturer’s protocols. The final DNA concentration and purification were determined by a NanoDrop 2000 UV-vis spectrophotometer (Thermo Scientific, United States), and DNA quality was checked by 1% agarose gel electrophoresis (Invitrogen Inc, United States). The V3-V4 hypervariable regions of the bacterial 16S rRNA gene were amplified with primers 338F (5′-ACT​CCT​ACG​GGA​GGC​AGC​AG-3′) and 806R (5′-GGACTACHVGGGTWTCTAAT-3′) by a thermocycler PCR system (GeneAmp 9700, ABI, United States). The PCR reactions were conducted using the following program: 3 min of denaturation at 95°C, 27 cycles of 30 s at 95°C, 30 s for annealing at 55°C, and 45 s for elongation at 72°C, and a final extension at 72°C for 10 min. PCR reactions were performed in triplicate 20 μl mixture containing 4 μl of 5 × FastPfu Buffer, 2 μl of 2.5 mM dNTPs, 0.8 μl of each primer (5 μM), 0.4 μl of FastPfu polymerase and 10 ng of template DNA. The resulting PCR products were extracted from a 2% agarose gel, further purified using the AxyPrep DNA Gel Extraction Kit (Axygen Biosciences, United States) and quantified using QuantiFluor™-ST (Promega, United States) according to the manufacturer’s protocol.

Purified amplicons were pooled in equimolar amounts and paired-end sequenced (2 × 300) on an Illumina MiSeq platform (Illumina, United States) according to the standard protocols by Majorbio Bio-Pharm Technology Co. Ltd (Shanghai, China). Raw fastq files were quality-filtered by Trimmomatic and merged by FLASH software with the following criteria: i) The reads were truncated at any site receiving an average quality score <20 over a 50 bp sliding window. ii) Sequences whose overlap being longer than 10 bp were merged according to their overlap with mismatch no more than 2 bp. iii)Sequences of each sample were separated according to barcodes (exactly matching) and Primers (allowing 2 nucleotide mismatching), and reads containing ambiguous bases were removed.

Operational taxonomic units (OTUs) were clustered with 97% similarity cutoff using UPARSE (version 7.1, http://drive5.com/uparse/) with a novel “greedy” algorithm that performs chimera filtering and OTU clustering simultaneously. The taxonomy of each 16S rRNA gene sequence was analyzed by RDP Classifier algorithm (http://rdp.cme.msu.edu/) against the Silva (SSU123) 16S rRNA database using confidence threshold of 70%.

The alpha diversity analysis reflected the abundance and diversity of a single sample microbial community. At the OTU level, the Sobs index was calculated to evaluate the species diversity of samples with different sequencing quantities. Linear discriminant analysis (LDA) was performed on samples of different groups to identify the species that had significant differences in sample classification using linear discriminate analysis effect size (LEfSe) software. One-way analysis of variance (ANOVA) was used to test the significance of the differences between groups. The copy number of the 16S marker gene in the species genome was removed using Phylogenetic Investigation of Communities by Reconstruction of Unobserved States software. The family information and Kyoto Encyclopedia of Genes and Genomes (KEGG) information from OTU Cluster of Orthologous Groups (COG) were obtained using the Greengenes ID corresponding to each OTU. Based on the information from the COG database, the descriptive information and functional information of each COG were analyzed from the Evolutionary Genealogy of Genes: Nonsupervised Orthologous Groups (egg NOG) database, and the functional abundance spectrum was obtained.

### Short-Chain Fatty Acid Analysis

Approximately 100 mg of fresh feces was precisely weighed, and 4 times methanol was added to fully homogenize the sample. The sample was centrifuged, and the supernatant was concentrated, then the residue was reconstituted with 50 μl of methanol for GC-FID analysis of SCFAs. The apparatus characteristics and chromatographic conditions are provided in Supplementary Method S2.

### Correlation Analysis Between Metabolites and Intestinal Flora

The Pearson correlation coefficient was used to express the relationships between various parameters, and a heat map was constructed using Pyplot. The correlation coefficient was always between − 1 and + 1, and the closer the absolute value of the correlation coefficient was to 1, the better the linear relationship.

### Statistical Analysis

SPSS 24.0 software and GraphPad Prism version 7.0 were used to analyze the data. The significant differences between multiple groups were calculated by one-way ANOVA. The unpaired two-tailed Student’s t-test was used between the two groups. If the *F*-test indicated that both populations had the same standard deviation (SD), then an unpaired *t*-test was used; otherwise, an unpaired *t*-test with Welch’s correction was used. The data are expressed as the mean ± SD, and a value of *p* < 0.05 indicated statistical significance.

## Results

### Qualitative and Quantitative Analysis of *Ziziphus jujuba* Mill. var. *spinosa* (Bunge) Hu ex H. F. Chou Seed Extract Composition

Based on the total ion chromatogram (Supplementary Figure S1) and mass spectrometry information (Supplementary Table S1), spinosin, 6‴-feruloylspinosin, jujuboside A, jujuboside B, betulinic acid, linoleic acid and oleic acid were identified in the ZSS extract compared to the literatures and reference compounds (Zhang et al., 2016; Wang et al., 2019), and their contents were quantified using HPLC-PDA-ELSD (Supplementary Figure S1 and Table S2). The results showed that the contents of spinosin, 6‴-feruloylspinosin, jujuboside A, jujuboside B, betulinic acid, linoleic acid, and oleic acid in the ZSS extract were 1.09, 0.61, 0.42, 0.20, 1.11, 0.73, and 1.77 mg/g, respectively (Supplementary Table S3).

### Model Verification and the Therapeutic Effect of *Ziziphus jujuba* Mill. var. *spinosa* (Bunge) Hu ex H. F. Chou Seed

**FIGURE 1:**
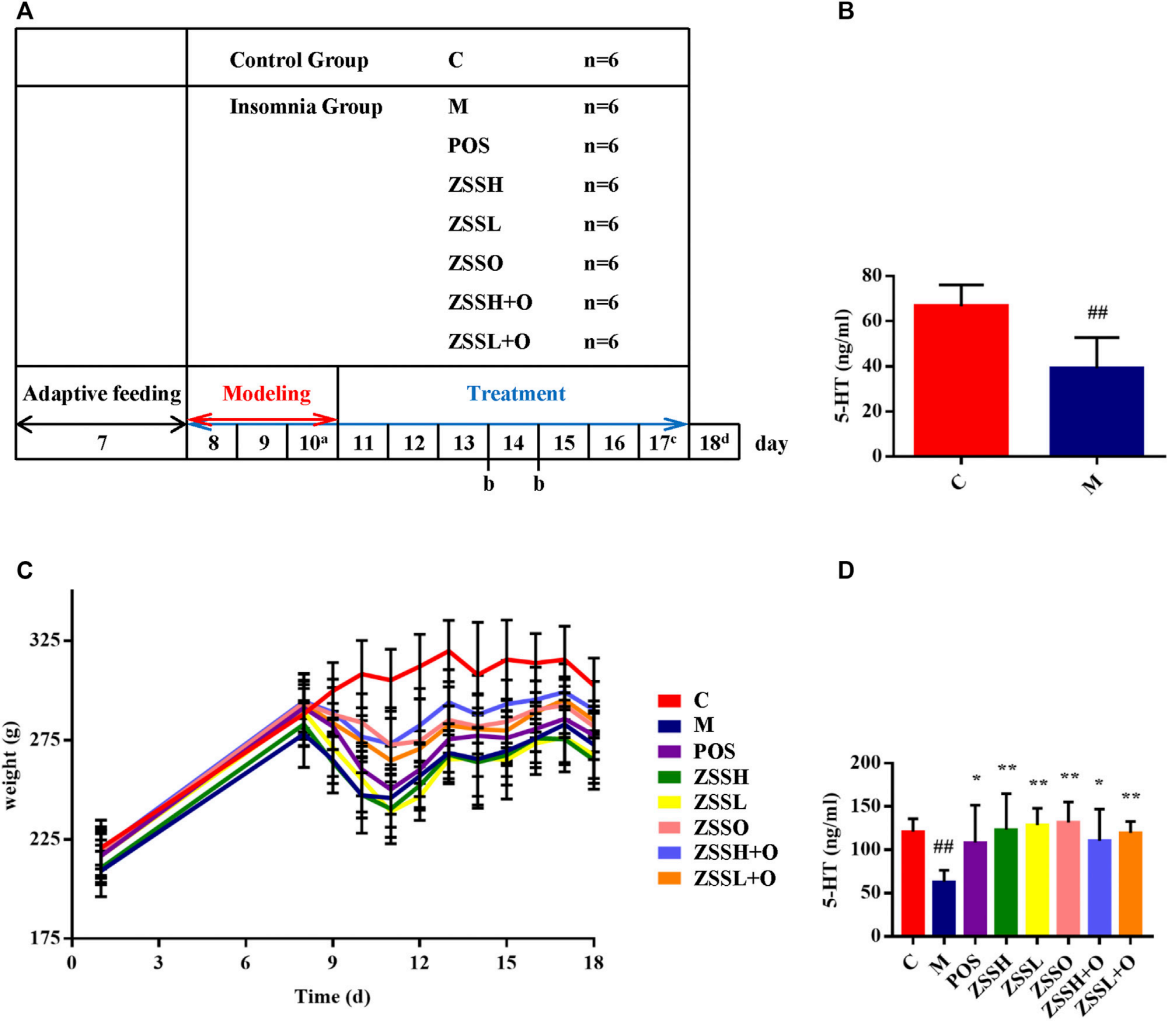
A schematic diagram for the time design of experiment **(A)**. a: Collecting C and M group plasma for model verification; b: Fasting for 12 h and collecting 12 h urine; c: Collecting fresh feces for 16S rRNA gene sequencing; d: Fasting for 12 h and collecting plasma. Determination of 5-HT among the C and M groups after modeling **(B)**. Weight changes during the experiment **(C)**. Determination of 5-HT after experiment **(D)**. (C) control group; (M) model group; (POS) positive group; (ZSSH) high-dose ZSS ethanol extract group; (ZSSL) low-dose ZSS ethanol extract group; (ZSSO) ZSS oil extract group; (ZSSH + O) high-dose ZSS ethanol + oil extract group; (ZSSL + O) low-dose ZSS ethanol + oil extract group. Values are presented as the mean ± SD, *n* = 6. #*p* < 0.05, ##*p* < 0.01: model vs control; *p < 0.05, **p < 0.01: treatment vs model.

The experimental process is shown in the schematic diagram (Figure 1A). After fasting for 12 h on the last day of modeling, blood was collected from the rat fundus venous plexus, and serum was separated. The results showed that the level of 5-HT in the serum of the model group was significantly lower than that of the control group (Figure 1B).

After the experiment, the model group still showed lower level of 5-HT than that of the control group, and the administration groups exhibited a significant callback (Figure 1D). In addition, except for the control group, the circadian rhythm of the other rats disappeared, indicating that the insomnia model was successful. During the modeling period, insomnia rats exhibited rough and dull hair, less sleep during the day, reduced diet, increased drinking, nonstop activity, manic restlessness, high sensitivity, excitability, and strong aggressiveness. Except for the control group, the food intake and weight of rats in each group decreased significantly during modeling. There were different degrees of callback after administration, and no significant difference was found between the administration and model groups (Figure 1C).

**FIGURE 2:**
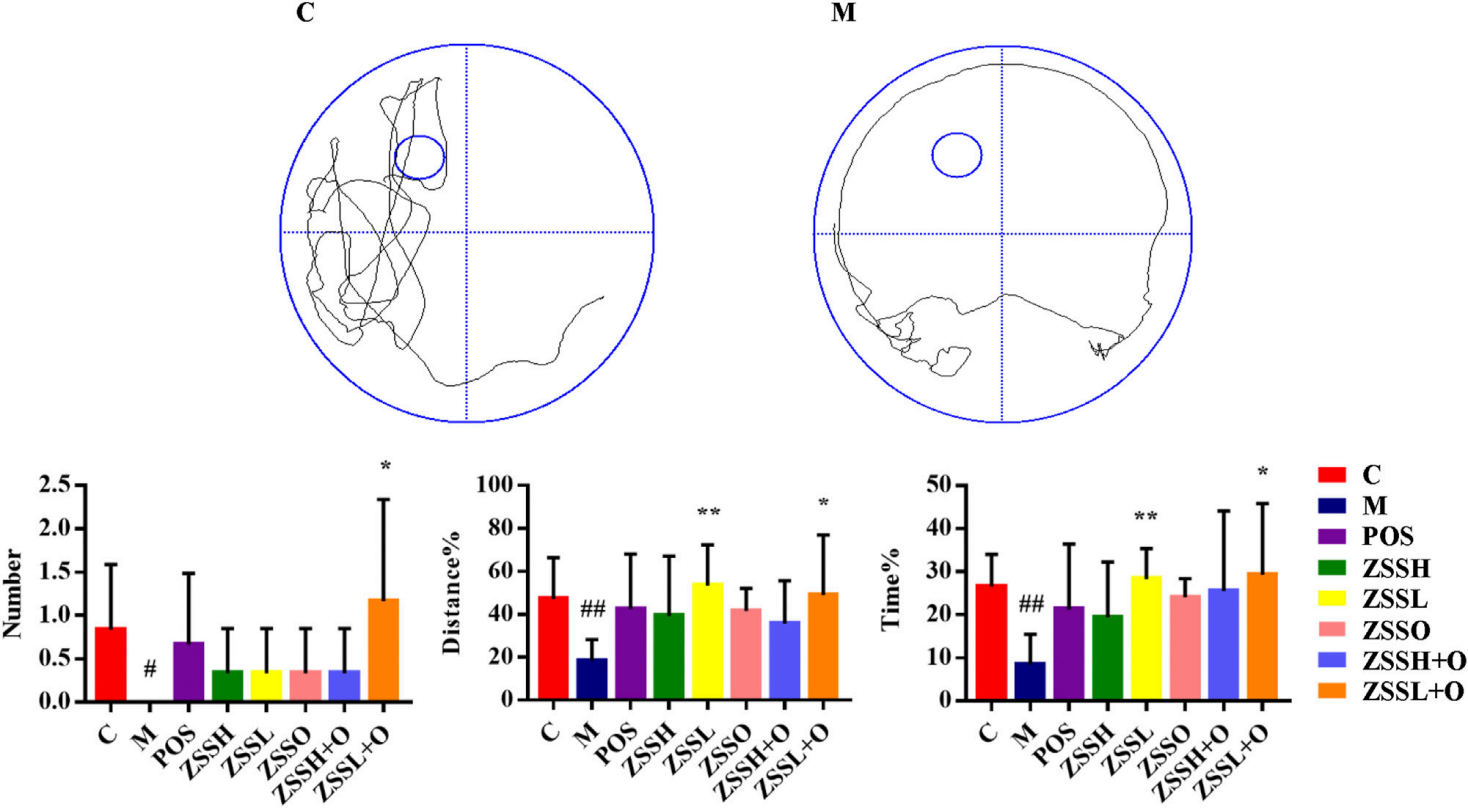
MWM evaluation indexes. (C) control group; (M) model group; (POS) positive group; (ZSSH) high-dose ZSS ethanol extract group; (ZSSL) low-dose ZSS ethanol extract group; (ZSSO) ZSS oil extract group; (ZSSH + O) high-dose ZSS ethanol + oil extract group; (ZSSL + O) low-dose ZSS ethanol + oil extract group. Values are presented as the mean ± SD, *n* = 6. #*p* < 0.05, ##*p* < 0.01: model vs control; *p < 0.05, **p < 0.01: treatment vs model.

To further investigate the therapeutic effect of ZSS, the MWM was used to test the learning and memory ability of insomnia rats. The multiple comparison results of one-way ANOVA showed that compared with the control group, the rats in the model group showed circling behavior, and the number of crossing platforms and the percentage of the distance and time to enter the target quadrant were significantly lower (Figure 2). After administration, the behavioral indexes trended to the levels of the control group, indicating that ZSS extract had a therapeutic effect on insomnia. The ZSSL and ZSSL + O groups had better therapeutic effects.

### Metabolism Analysis

**FIGURE 3:**
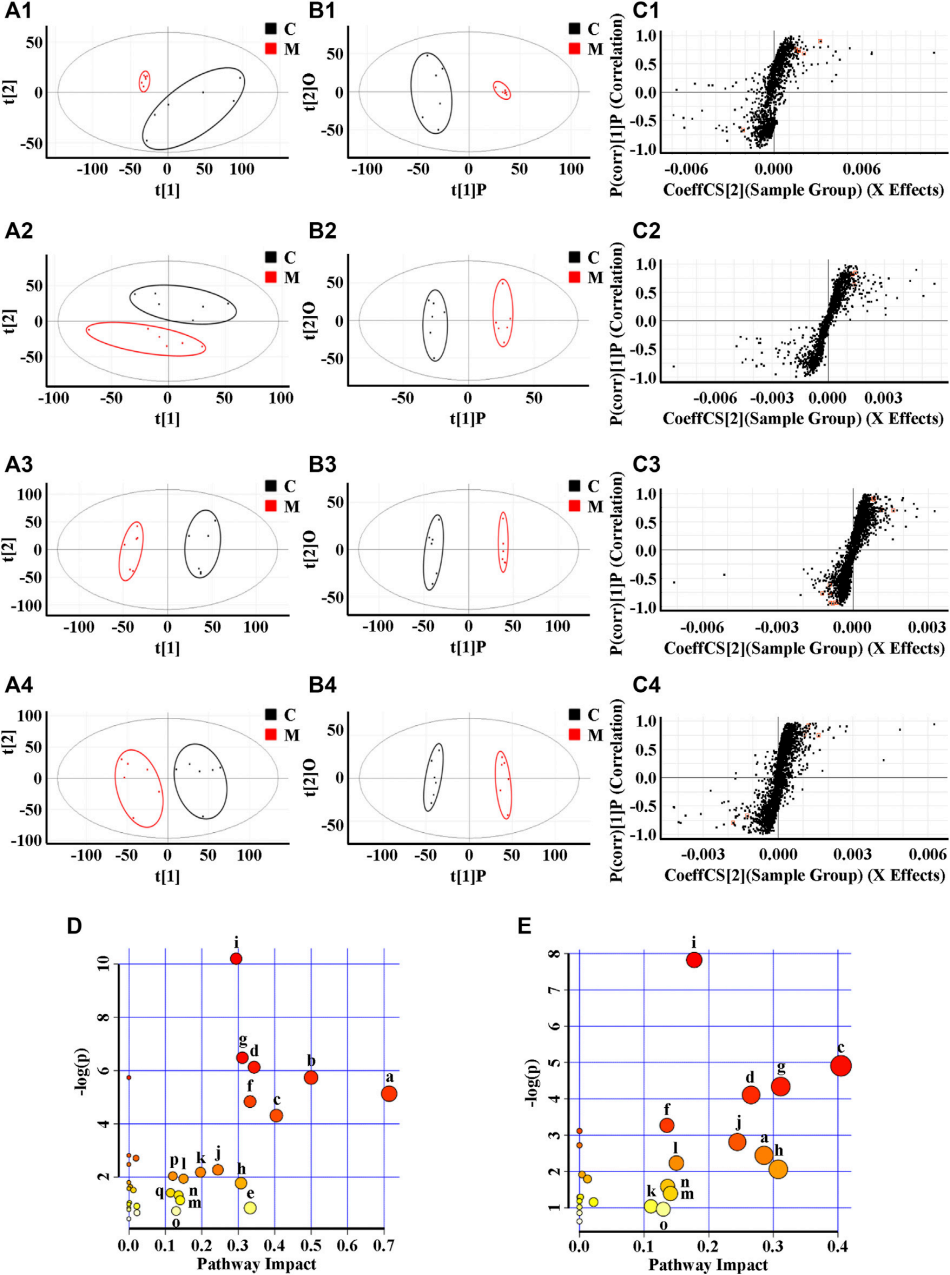
PCA **(A)**, OPLS-DA **(B)** and S-plot of OPLS-DA **(C)** for plasma (1, 2) and urine (3, 4) of model group (red) vs. control group (black) in positive (1, 3) and negative (2, 4) ion mode; metabolic pathways involved in all markers in plasma and urine for insomnia **(D)**; metabolic pathways involved in potential markers in plasma and urine regulated by ZSS **(E)**. (a) Taurine and hypotaurine metabolism; (b) D-glutamine and D-glutamate metabolism; (c) phenylalanine metabolism; (d) pentose and glucuronate interconversions; (e) arachidonic acid metabolism; (f) alanine, aspartate and glutamate metabolism; (g) histidine metabolism; (h) caffeine metabolism; (i) arginine biosynthesis; (j) glycerophospholipid metabolism; (k) arginine and proline metabolism; (l) biotin metabolism; (m) lysine degradation; (n) selenocompound metabolism; (o) tyrosine metabolism; (p) one carbon pool by folate; (q) terpenoid backbone biosynthesis.

**FIGURE 4:**
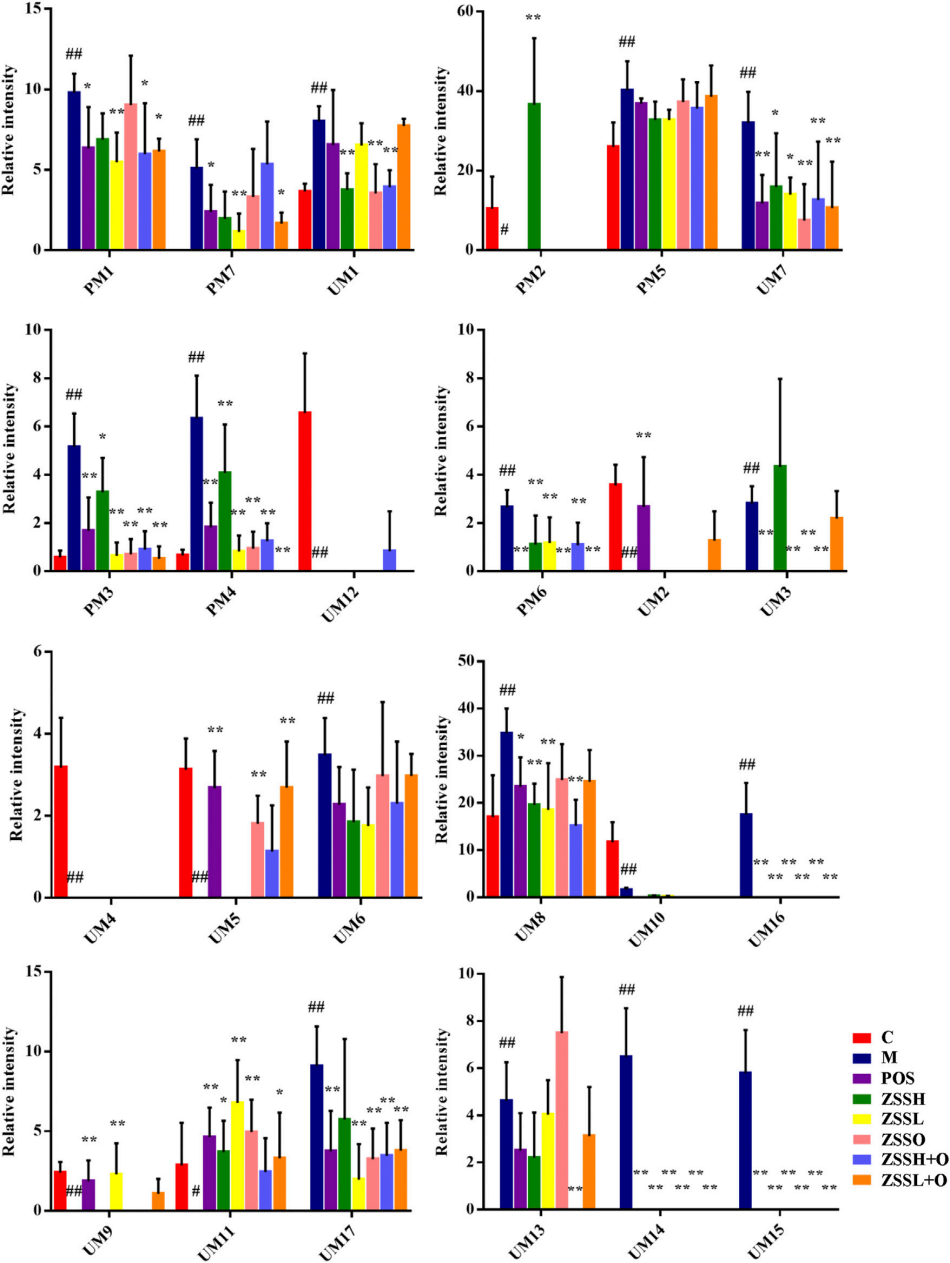
Relative peak areas of potential biomarkers identified in plasma and urine in positive and negative ion modes. (C) control group; (M) model group; (POS) positive group; (ZSSH) high-dose ZSS ethanol extract group; (ZSSL) low-dose ZSS ethanol extract group; (ZSSO) ZSS oil extract group; (ZSSH + O) high-dose ZSS ethanol + oil extract group; (ZSSL + O) low-dose ZSS ethanol + oil extract group. Values are presented as the mean ± SD, *n* = 6. #*p* < 0.05, ##*p* < 0.01: model vs control; *p < 0.05, **p < 0.01: treatment vs model.

Due to the limitation of sample collection and time arrangement, rats’ urine was collected in parallel for 12 h according the references (Giri et al., 2007; Zhou et al., 2011). The mass spectrum data were normalized by total peak area (Gardlo et al., 2016). The metabolic information of plasma and urine detected by UPLC-QTOF/MS were analyzed using PCA. The PCA score plots (Figure 3(A1)–(A4)) showed significant clustering in plasma and urine samples of the C and M groups in both positive and negative ion modes, indicating that the samples from the M group deviated from normal levels and showed metabolic disorders. Subsequently, potential markers of interest (marked in red boxes) were extracted from the S-plots (Figure 3(C1)–(C4)) constructed after OPLS-DA (Figure 3(B1)–(B4)). The metabolites in plasma and urine samples were identified based on MS/MS data, and a total of 24 metabolites were annotated (7 metabolites from plasma and 17 metabolites from urine). The mass spectrometry data and their changing trends in the model group compared to the control group are shown in Supplementary Table S4. Among the 24 metabolites, the levels of nine metabolites, including PE (22:2 (13Z,16Z)/P-18:1 (11Z)), taurine, L-glutamic acid, L-glutamine, 1-methylxanthine, 5,10-methenyltetrahydrofolic acid, D-xylulose, D-xylose, and urocanic acid, significantly decreased in the model group compared to the control group, whereas the other 15 metabolite levels dramatically increased. The relative contents of 24 potential biomarkers in each group were analyzed using one-way ANOVA, and the differences between the model group and other groups were compared. The results (Figure 4) showed that the contents of 18 biomarkers were reversed (*p* < 0.05) after administration of ZSS extract, except for arachidonic acid (PM5) in plasma and taurine (UM2), L-glutamic acid (UM4), mevalonic acid (UM6), 5,10-methenyltetrahydrofolic acid (UM10) and D-xylose (UM12) in urine. These results indicated that the administration of ZSS extract could effectively regulate the abnormal changes in these potential biomarkers.

To explore the possible pathways influenced by the ZSS extract, the metabolites identified in plasma and urine were analyzed using the MetaboAnalyst database to construct metabolic pathways. As shown in Figure 3D, the larger the-log (P) and the redder the color, the higher the significance of the metabolites, and the larger the pathway impact. The pathway with an impact value greater than 0.1 was screened as the potential target pathway. The results showed that the occurrence of insomnia might be associated with multiple pathways, among which the top ten metabolic pathways include (a) taurine and hypotaurine metabolism; (b) D-glutamine and D-glutamate metabolism; (c) phenylalanine metabolism; (d) pentose and glucuronate interconversions; (e) arachidonic acid metabolism; (f) alanine, aspartate and glutamate metabolism; (g) histidine metabolism; (h) caffeine metabolism; (i) arginine biosynthesis; and (j) glycerophospholipid metabolism. Phenylalanine metabolism (c) was considered the most important metabolic pathway modified by ZSS extract, with an impact value of 0.40476 (Figure 3E), suggesting that the administration of ZSS extract could modify this metabolic pathway and related biomarkers to exert a curative effect on insomnia.

### Intestinal Flora Analysis

**FIGURE 5:**
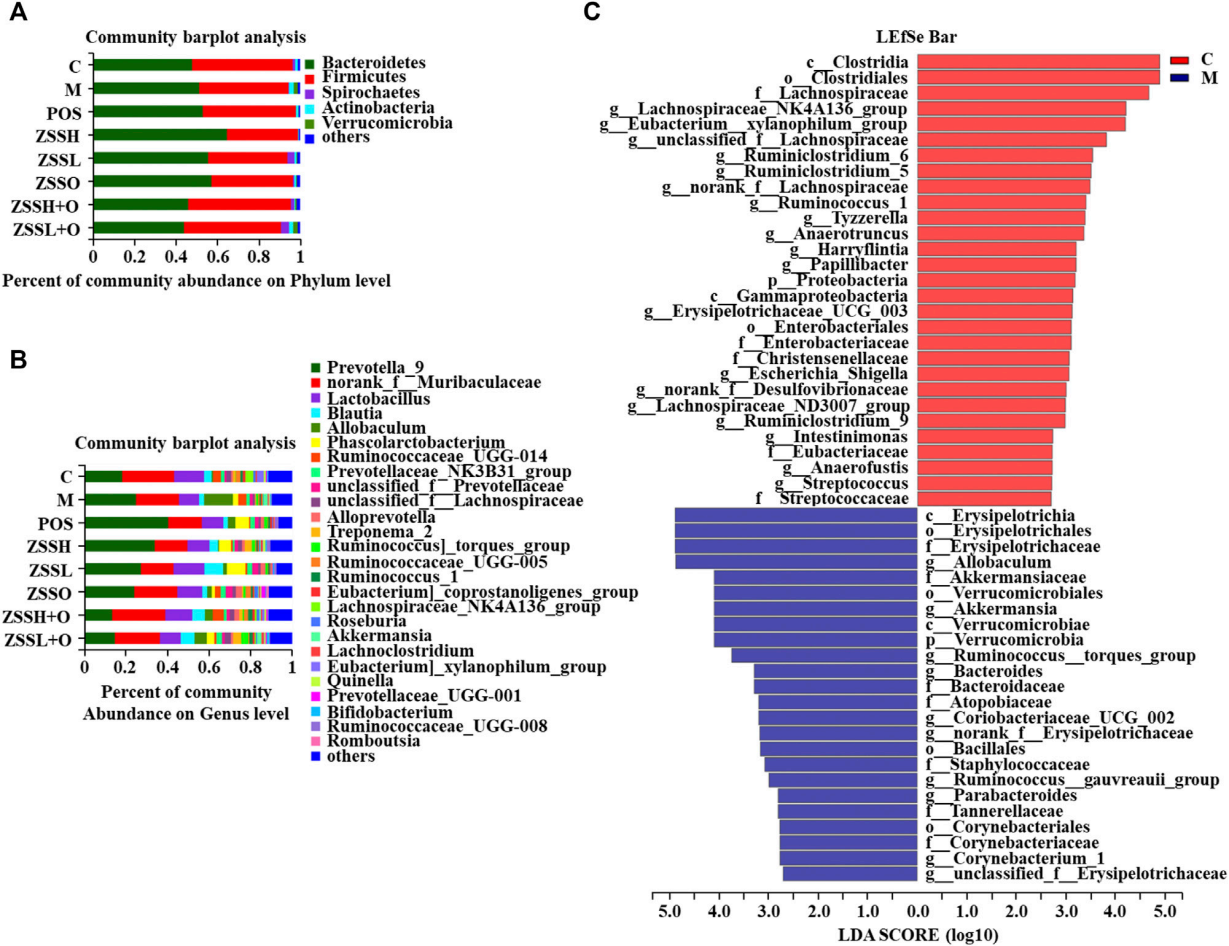
Effects of ZSS extract on the structure and abundance of intestinal flora in insomnia rats. The percent of community abundance at the phylum **(A)** and genus **(B)** levels. LDA scores **(C)** were generated from LEfSe analysis, showing the differences in bacterial abundance at the genus level.

To explore whether the sedative and hypnotic effects of ZSS extract were associated with intestinal flora, 16S rRNA gene sequences of the rat’s fecal flora after treatment were analyzed, and the species diversity of the samples was evaluated at the OTU level. The curves of the Sobs exponent (Supplementary Figure S2(A)) indicated that the microflora of each group had a high richness, and there was no significant difference between the different groups. The histograms of the community (Figure 5A,B) showed that after combining species with low abundance, the relative abundances of *Bacteroides* and *Firmicutes* at the phylum level and *Prevotella_9* and *norank_f__Muribaculaceae* at the genus level were higher. Compared to the control group, the community composition diversity changes of the model group were mainly as follows: at the phylum level, *Actinobacteria* and *Verrucomicr obia* in the model group increased, and *Spirochaetes* decreased; while at the genus level, *Allobaculum* increased considerably. Significant differences between the model group and the control group were identified using LEfSe analysis (Figure 5C). From the phylum level to the genus level, there were 53 significant differences, while 27 were significant at the genus level. At the genus level, *Lachnospiraceae_NK4A136_group* and *Eubacterium_xylanophilum_group* had the strongest association with the control group, while *Allobaculum* and *Akkermansia* were the most representative bacteria in the model group.

**FIGURE 6:**
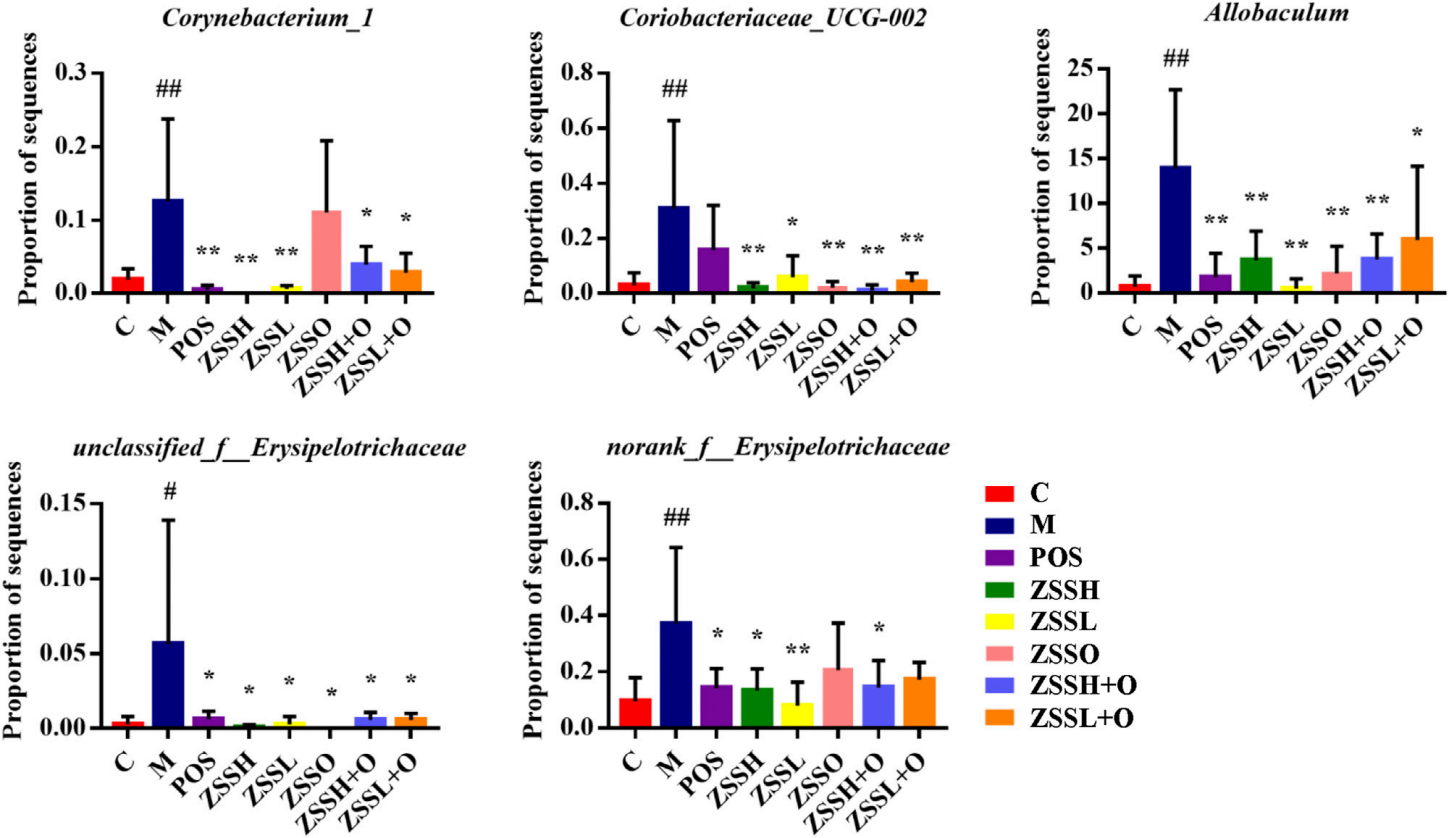
Average relative abundance of different genera of intestinal flora in each group. (C) control group; (M) model group; (POS) positive group; (ZSSH) high-dose ZSS ethanol extract group; (ZSSL) low-dose ZSS ethanol extract group; (ZSSO) ZSS oil extract group; (ZSSH + O) high-dose ZSS ethanol + oil extract group; (ZSSL + O) low-dose ZSS ethanol + oil extract group. Values are presented as the mean ± SD, *n* = 6. #*p* < 0.05, ##*p* < 0.01: model vs control; *p < 0.05, **p < 0.01: treatment vs model.

Based on one-way ANOVA, the average relative abundance of different genera of the intestinal flora was calculated, and it was found that the levels of five genera, including *Corynebacterium_1*, *Coriobacteriaceae_UCG-002*, *unclassified_f__Erysipelotrichaceae*, *norank_f__Erysipelotrichaceae*, and *Allobaculum,* were reversed to normal levels after administration of ZSS extract (Figure 6). Additionally, a total of 24 metabolic functions were found to be related to the intestinal flora of insomnia rats (Supplementary Figure S2(B)). Among them, carbohydrate transport and metabolism, DNA replication, recombination and repair, and amino acid transport and metabolism were the most important COG functions. Based on the information from the KEGG database, the top 20 metabolic pathways (Supplementary Table S5) were screened out, in which carbohydrate, amino acid, energy, cofactors and vitamins, nucleotide, lipid, and glycan biosynthesis metabolism and other metabolic pathways contributed directly or indirectly to the development of insomnia.

### Short-Chain Fatty Acid Analysis

**FIGURE 7:**
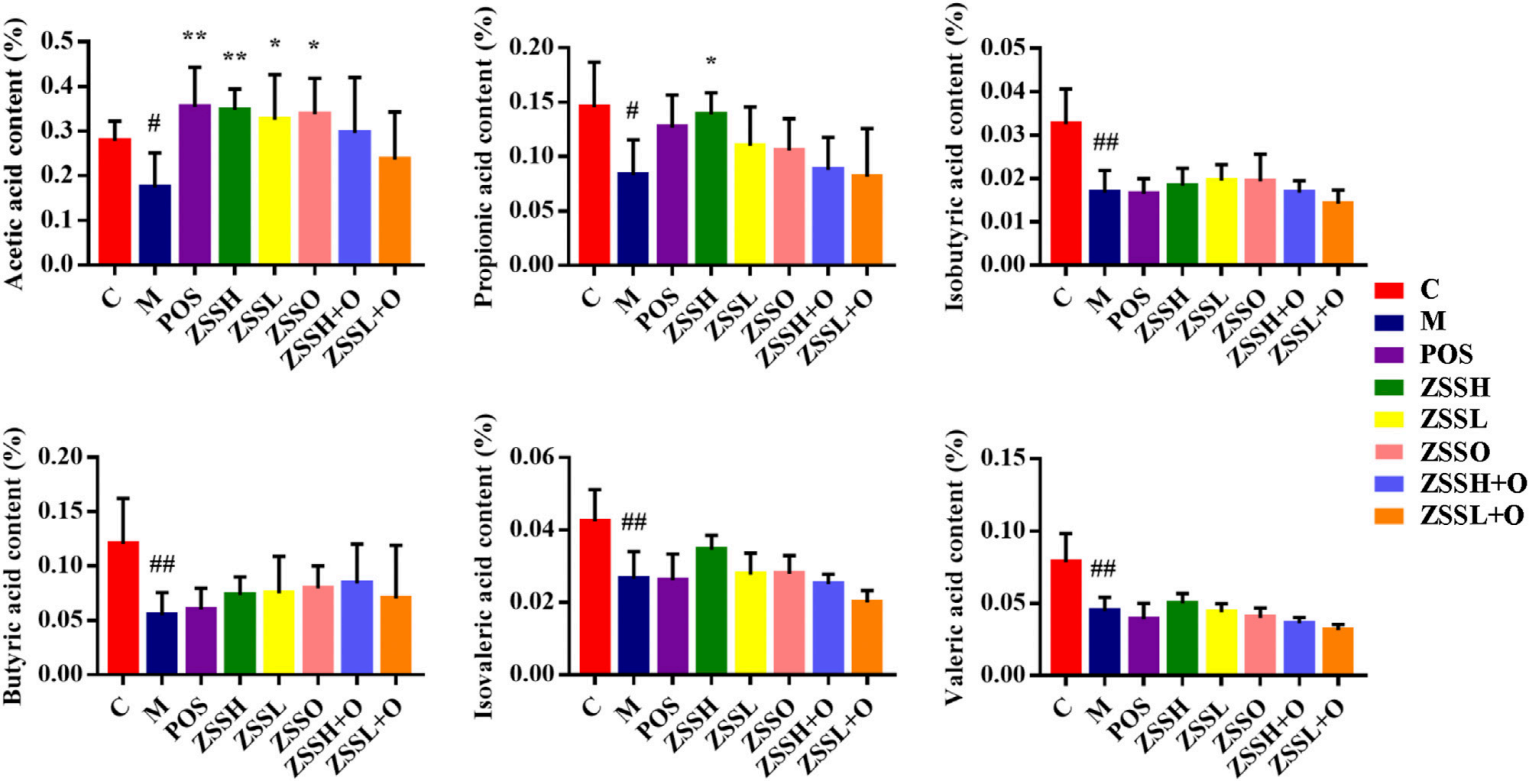
Effects of ZSS components on SCFAs in feces. (C) control group; (M) model group; (POS) positive group; (ZSSH) high-dose ZSS ethanol extract group; (ZSSL) low-dose ZSS ethanol extract group; (ZSSO) ZSS oil extract group; (ZSSH + O) high-dose ZSS ethanol + oil extract group; (ZSSL + O) low-dose ZSS ethanol + oil extract group. Values are presented as the mean ± SD, *n* = 6. #*p* < 0.05, ##*p* < 0.01: model vs control; *p < 0.05, **p < 0.01: treatment vs model.

The contents of six SCFAs (acetic acid, propionic acid, isobutyric acid, butyric acid, isovaleric acid and valeric acid) in the feces of insomnia rats were determined using GC-FID (Supplementary Table S6-7 and Figure S3). Dunnett’s multiple comparisons test showed that the contents of SCFAs in the model group were significantly lower than those in the control group, and there was a tendency toward callback after administration of ZSS extracts (Figure 7). Among them, the reverse effect of acetic acid and propionic acid was more significant.

### Potential Connections Between Plasma and Urine Metabolites and Intestinal Flora

**FIGURE 8:**
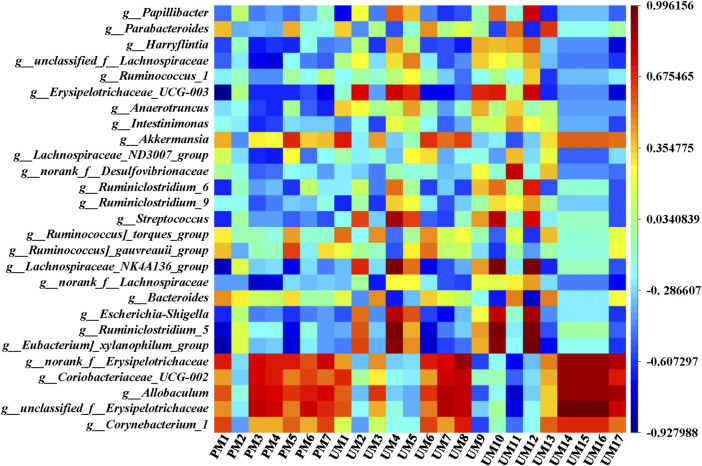
Correlation analysis between metabolites and intestinal flora.

To comprehensively analyze the relationships between 24 metabolites and 27 genera of the intestinal flora, a correlation matrix was established by calculating the Pearson correlation coefficient, as shown in Figure 8. Regarding the relationships between metabolites and intestinal flora, the five genera with significant callback after administration of ZSS extracts, including *Corynebacterium_1*, *unclassified_f__Erysipelotrichaceae*, *Allobaculum*, *Coriobacteriaceae_UCG-002*, and *norank_f__Erysipelotrichaceae*, were negatively correlated with 1-methylxanthine (UM9) and D-xylulose (UM11), while they were positively correlated with most metabolites, especially phenylacetaldehyde (UM14), urocanic acid (UM15), phenylpyruvic acid (UM16), and aminoadipic acid (UM8). Moreover, *Eubacterium]_xylanophilum_group*, *Ruminiclostridium_5*, *Escherichia-Shigella*, *Lachnospiraceae_NK4A136_group*, *Streptococcus*, and *Erysipelotrichaceae_UCG-003* were positively correlated with L-glutamic acid (UM4), 5,10-methenyltetrahydrofolic acid (UM10), and D-xylose (UM12), while they were negatively correlated with 3-sulfinoalanine (PM1). *Bacteroides* and UM4, UM10, UM12, *norank_f__Lachnospiraceae and* 6-dehydrotestosterone glucuronide (PM3), 15-hydroxynorandrostene-3,17-dione glucuronide (PM4) were negatively correlated. These relationships suggested that metabolites and intestinal flora were closely related and could influence each other.

## Discussion

Insomnia is the most common type of sleep disorder in the clinic. According to the theory of Western medicine, PCPA, which is a 5-HT inhibitor, is often used to establish a sleep deprivation animal model by intraperitoneal injection to study the pharmacological mechanism of certain hypnotic drugs (Yang et al., 2020). The method is easy to operate and does not require special equipment, but there are still individual differences in experimental animals.

It was reported that chemical constituents in ZSS, including saponins, flavonoids, and alkaloids, exert sedative and hypnotic effects primarily through the *γ*-aminobutyric acid (GABA)-ergic and serotonergic systems. Different from water extraction of TCM, ethanol extraction was used in this paper because most of the effective components (such as saponins and flavonoids) in ZSS have good solubility in organic solvents, and ethanol extract has better sedative and hypnotic effects than water extract for ZSS (Jiang et al., 2007). We speculatively believed that changing the extraction method with ethanol won’t change the traditional use of Chinese medicine, but will make its pharmacological effect better. Additionally, most of the research for ZSS treating insomnia ignored the fatty oil which is abundant in ZSS. Thus, ZSS was extracted with petroleum ether to obtain ZSS oil first and then extracted with ethanol in this study, and the animals were divided into ZSS ethanol extract, ZSS oil extract and combination groups to explore whether ZSS oil can promote the sedative effects of ZSS. The results showed that ZSS oil also had a certain effect on insomnia, but the effect when it combined with ethanol extract was not significantly different from that of ethanol extract administration alone, indicating that fatty oil did not play an important role in the sedative effect of ZSS. This may explain why the traditional extraction techniques for ZSS ignoring fatty oil.

In this experiment, by measuring the 5-HT content and behavioral indicators of insomnia rats during this process, it was proven that the insomnia model was successfully implemented. At the same time, the behavioral indexes of the MWM confirmed that insomnia also had a certain degree of influence on the learning and memory ability of rats. However, there are still some procedures need to be improved in the MWM experiment. The insomnia rats could restore gradually as time goes on (Gao et al., 2002), which leads to some limitations in the design of experimental time. The water maze time arrangement used less time for acquisition and probe trials, which was not strict enough and was only for reference. These results indicate that ZSS has a good therapeutic effect on insomnia.

Till now, few reports show side effects of BZD or Selective Serotonin Reuptake Inhibitor (SSRI)-related drugs for ZSS. However, a Meta-analysis of the modified Suanzaoren Decoction in the treatment of insomnia showed the side effects, such as dizziness, headache, abdominal distension, nausea, constipation and fatigue (Wang et al., 2018). Due to ZSS is an important component of Suanzaoren Decoction, the result suggests that whether ZSS has the above side effects is worth further study.

This study aimed to evaluate the therapeutic effect of ZSS extract and to elucidate its anti-insomnia mechanism by metabolomics and intestinal flora profiles. Therefore, the metabolomics, intestinal flora, and their correlations in insomnia rats were studied according to the expected assumptions. Metabolomics explains physiological and pathological conditions and reflects the rule that subjects are affected by internal and external factors in their overall metabolism by analyzing the composition and changes in endogenous metabolites. Results showed that the top five metabolic pathways related to insomnia were as follows: taurine and hypotaurine metabolism (Du et al., 2019b; Yang et al., 2020), D-glutamine and D-glutamate metabolism (Du et al., 2019b), phenylalanine metabolism (Du et al., 2020), pentose and glucuronate interconversions, and arachidonic acid metabolism (Liu et al., 2019). A recent review (Humer et al., 2020) of metabolomics in insomnia suggested that sleep-wake disorders lead to pronounced alterations in specific metabolic pathways, such as amino acid and glucose metabolism, which is basically consistent with the results of metabolism research in this study. In addition, elevated levels of the branched-chain amino acids including isoleucine, leucine and valine were found to be associated with later sleep timing for humans (Xiao et al., 2017; Humer et al., 2020), while which was not found in this study for the insomnia rats. Whether there are species differences needs further study.

Metabolic analysis results also demonstrated that ZSS extract could modify the levels of the vast majority of differential metabolites and improved insomnia by mainly regulating the following five metabolic pathways: phenylalanine metabolism, histidine metabolism (Yang et al., 2020), caffeine metabolism, taurine and hypotaurine metabolism, and pentose and glucuronate interconversions.

Among the metabolic pathways found in this study, phenylalanine metabolism was the most important metabolic pathway regulated by ZSS extract. Phenylalanine is an essential amino acid and also the precursor of tyrosine. Like tyrosine, phenylalanine is also a precursor for catecholamines, including tyramine, dopamine, epinephrine, and norepinephrine. These excitatory neurotransmitters participate in the regulation of the sleep-wake cycle (Du et al., 2020). The level of phenylalanine in the insomnia model rats increased under stress. Excessive phenylalanine led to the increase of downstream excitatory neurotransmitters, which made rats awake and insomnia. It can also be metabolized into phenylpyruvate through a transaminase pathway route involving glutamate. Therefore, the increase in the downstream metabolites, such as phenylacetaldehyde, phenylpyruvic acid and dopamine, also proved that phenylalanine metabolism was abnormal.

Histamine, as an important neurotransmitter in the brain, is converted from histidine by histidine decarboxylase (Yang et al., 2020). There is evidence in both animals and humans that histamine release is a key element in maintaining wakefulness (Haas and Panula, 2003). Histamine is involved in the regulation of complex behaviors that are controlled by several neurotransmitter systems, such as dopamine, GABA, and glutamate. It is associated with neuroinflammation, brain damage, headaches (Haas et al., 2008) and various central nervous diseases, such as Alzheimer’s disease, Down’s syndrome, Parkinson’s disease, and schizophrenia. Urocanic acid, as an intermediate in the metabolic pathway from histidine to glutamate, is produced by intramolecular anaerobic deamination under the action of histidase. Usually, urocanic acid is further decomposed into glutamic acid under the action of urocanase. Both histamine and urocanic acid are converted from histidine, and the content of urocanic acid decreased in the model group, while the content of histamine increased, indicating that most histidine is converted into histamine, which is closely related to the poor sleep behaviors of insomnia rats during the day. After administration of ZSS, the metabolite levels returned to normal, therefore, one of the main ways in ZSS regulating insomnia is through histidine metabolism.

The level of 3-sulfinoalanine in the plasma of model rats increased. It is known that 3-sulfinoalanine can promote the formation of taurine, and interacts with GABA to activate GABA receptors and glycine receptors to further strengthen the inhibition of the central nervous system (Yang et al., 2020). Taurine is an inhibitory neurotransmitter, and its content increased in the plasma (Davies et al., 2014) and cerebral cortex (Mohammed et al., 2011) of sleep-deprived rats. But it is shown that the excretion of taurine in urine decreased in this study. It is believed that the insomnia can cause the disorder of taurine and hypotaurine metabolism in rats.

Caffeine is a most widespread psychostimulant, and its main physiological effect is to stimulate the central nervous system. 1-Methylxanthine is a major metabolite of caffeine. The excretion of 1-methylxanthine in the urine of model group was reduced, indicating that caffeine accumulation in the body caused insomnia. 6-Dehydrotestosterone glucuronide and 15-hydroxynorandrostene-3,17-dione glucuronide are β-D-glucuronosides which are generated in the liver by UDP glucuronyltransferase. Glucuronidation is used to assist the excretion of toxic substances, drugs or other substances that cannot be used as energy sources (Sun et al., 2018). D-Xylulose is converted from xylitol by the enzyme NAD + -linked xylitol dehydrogenase in the glucuronate pathway, the most important xylitol-handling metabolic pathway in mammals. *β*-D-glucuronoside in the plasma increased, while D-xylulose in the urine decreased, suggesting that insomnia increased the levels of toxic substances in the body. The abovementioned metabolic changes indicated that caffeine metabolism and pentose and glucuronate interconversions were abnormal in insomnia rats and could be adjusted by ZSS extract.

In conclusion, the results of metabolomics showed that ZSS can intervene insomnia by regulating a variety of metabolic pathways related to insomnia. This intervention is mostly related to several key components in the pathways, which is mainly reflected in regulating the abnormal neurotransmitters, alleviating the neuroinflammation and reducing the accumulation of toxic components in the body. Based on the above conclusions, we would give metabolites related to the regulation of insomnia metabolic disorder, so as to further verify its mechanism of action in the body.

To understand and use ZSS more effectively, the interactions between ZSS and intestinal flora composition were also studied. Based on 16S rRNA gene amplicon sequencing, it was demonstrated that between insomnia and healthy rats, the composition, diversity, and metabolic function of the intestinal flora are significantly changed. By comparing the differences in relative abundance, we identified that *Bacteroidetes* were the dominant taxa in the insomnia group, while *Firmicutes* was enriched in the control group, resulting in a decreased ratio of *Firmicutes/Bacteroidetes* in model group, which is consistent with the report on the intestinal flora of insomnia humans (Liu et al., 2019). Analysis of the fecal microbiota showed that the abundances of the genera *Corynebacterium_1* and *Coriobacteriaceae_UCG-002* (family *Coriobacteriaceae*, phylum *Actinobacteria*), *unclassified_f__Erysipelotrichaceae*, *norank_f__Erysipelotrichaceae*, and *Allobaculum* (family *Erysipelotrichaceae*, phylum *Firmicutes*) in the intestinal flora of insomnia rats were significantly increased compared to those in control rats.

Among them, the abundance of *Corynebacterium_1* was previously demonstrated to be correlated with serum concentrations of interleukin-6 and C-reactive protein in cancer patients (Ning et al., 2020). Therefore, insomnia is not only related to metabolism but also an autoinflammatory disease associated with an increased inflammatory reaction, which suggests an association between intestinal flora such as *Corynebacterium_1* and an inflammatory reaction. Meanwhile, *Erysipelotrichaceae* is associated with several diseases, such as inflammation-related intestinal disease and metabolic disorders in humans (Kaakoush, 2015). It is also related to high-fat diets in humans and rodent models (Martinez et al., 2009). *Erysipelotrichaceae* may play a central role in the relationships between microbiota, macronutrient composition and digestibility, fecal health score and fecal weight (Bermingham et al., 2017). It appears to be an important bacterium in the digestive tract, and an increase in *Erysipelotrichaceae* is mainly caused by an increase in the number of *unclassified_f__Erysipelotrichaceae*, *norank_f__Erysipelotrichaceae*, and *Allobaculum*. The specific phylotype *Allobaculum* which is also a SCFA-producing bacterium (Zhang et al., 2012), was observed to be enriched after exercise (Petriz et al., 2014; Campbell et al., 2016). This may be related to less sleep, more activity, high excitability, and aggressiveness in insomnia rats. Based on the KEGG database, the carbohydrate, amino acid, energy metabolism and others contributed directly or indirectly to the development of insomnia.

SCFAs are produced by beneficial bacteria in the microbiome. They are believed to have many important roles in maintaining body health, such as protecting the intestinal mucosal barrier, reducing the level of inflammation, and enhancing gastrointestinal motility (Rios-Covian et al., 2016). In this study, SCFAs in the feces decreased in the model group, indicating that insomnia would reduce SCFAs, increase inflammation, and then affect intestinal health. In this study, the SCFAs in insomnia rats decreased, while the SCFA-producing bacteria in the intestinal flora increased instead, which indicates a certain contradiction. During the modeling period, the insomnia rats had less food, and their weight decreased significantly; after the modeling was completed, their food intake gradually increased and exceeded that of the control group, and the weight also gradually increased. Based on this contradictory behavior in rats, it is speculated that the lack of SCFAs has a negative feedback effect on SCFA-producing bacteria. To make the SCFA deficiency in insomnia rats return to normal levels, the diet and SCFA-producing bacteria increased, which led to this contradiction. A variety of intestinal flora associated with insomnia and the changes of SCFA contents showed that insomnia was closely related to inflammatory reaction, food intake and exercise.

Potential connection analysis between metabolites and intestinal flora showed that the key intestinal flora of ZSS regulating insomnia was closely related to the metabolites adjusting insomnia. Metabolomics studies have shown that the metabolic pathways associated with insomnia were mostly related to amino acid metabolism (mainly taurine and hypotaurine metabolism, D-glutamine and D-glutamate metabolism, phenylalanine metabolism, alanine, aspartate and glutamate metabolism, histidine metabolism, arginine biosynthesis, arginine and proline metabolism, lysine degradation, and tyrosine metabolism), which was consistent with the amino acid transport and metabolism in the prediction of intestinal flora function. The increase in specific intestinal flora, the decrease in SCFAs, and the metabolic abnormality of the arachidonic acid metabolism and other pathways all proved that insomnia is related to inflammation. This result further indicated that insomnia was a synergistic effect of metabolism and intestinal flora, and ZSS can improve insomnia by adjusting metabolic profiles and intestinal flora composition.

Since the active ingredients in ZSS couldn’t pass through the blood-brain barrier, its mechanism of action on insomnia is still unclear. This study used another possible mechanism to analyze the effect of ZSS on intestinal flora, which is “brain-gut-microbiota axis”, indicating the intestinal flora might affect the brain function. The results showed that insomnia is related to multiple metabolic pathways and intestinal flora, and ZSS extract can regulate metabolic profiles and improve the composition of intestinal flora in insomnia rats. This may be related to the regulation of metabolic disorders through amino acid metabolism, as well as the reversal of intestinal flora disorders and SCFA abnormalities caused by insomnia. Previous studies have shown that the improvement of insomnia by ZSS is related to amino acid metabolism. This paper is another verification of previous research conclusion. Besides, this study explored the sedative and hypnotic effects of ZSS from the perspective of metabolomics and intestinal flora, which could provide useful ideas for the study of the mechanism of insomnia and contribute to the utilization of ZSS resources.

## Data Availability

The datasets generated in this study have been deposited to an online repository. This data can be found at https://www.ncbi.nlm.nih.gov/sra using the accession number PRJNA684956.
