# Supplementary figures and images for: Ziziphus jujuba Mill. var. spinosa (Bunge) Hu ex H. F. Chou Seed Ameliorates Insomnia in Rats by Regulating Metabolomics and Intestinal Flora Composition
Source: Front Pharmacol. 2021 Jun 16;12:653767. doi: 10.3389/fphar.2021.653767 (PMC8241942; doi:10.3389/fphar.2021.653767)

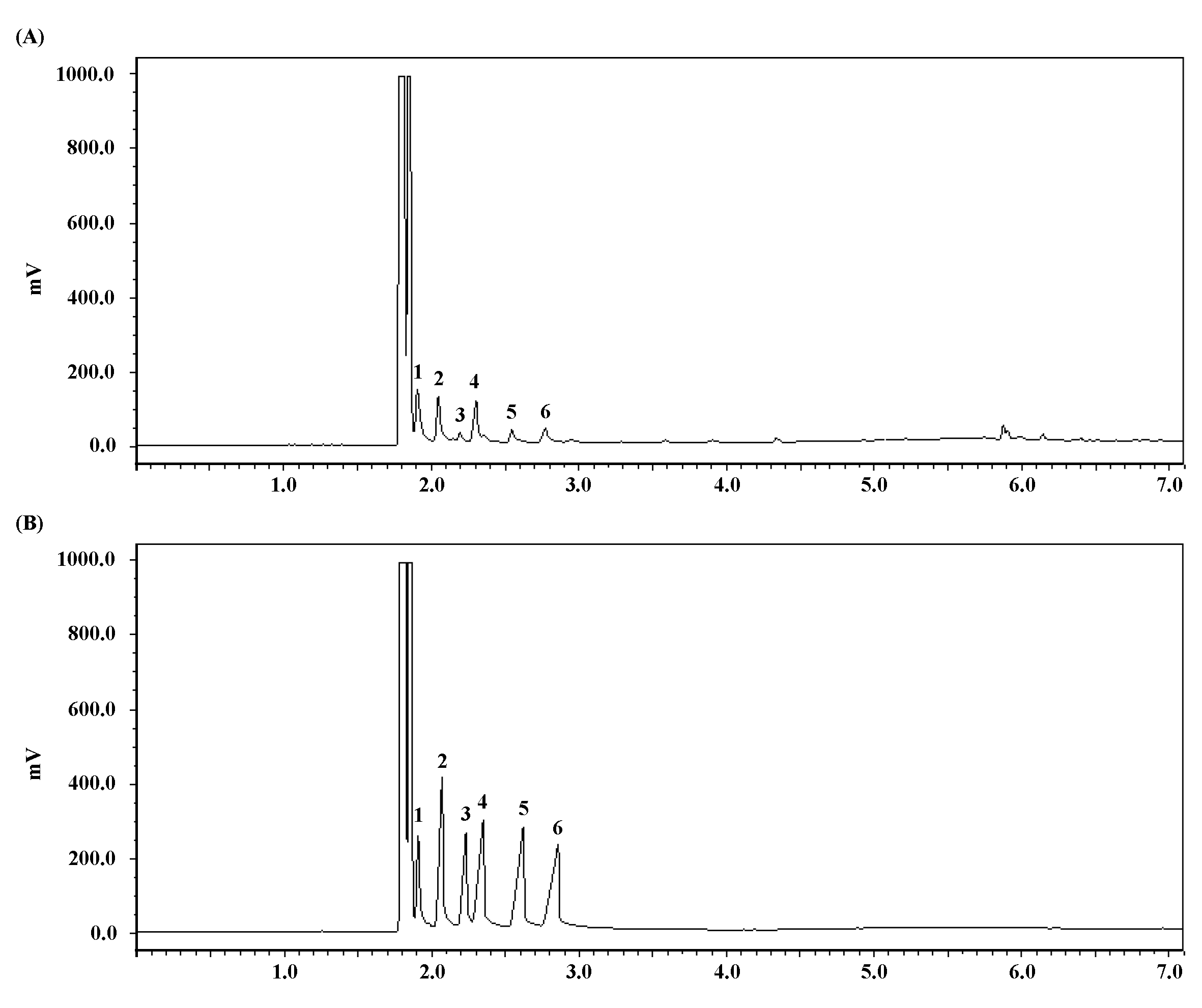

Supplement: Supplementary file 1 [file Image3.TIFF]

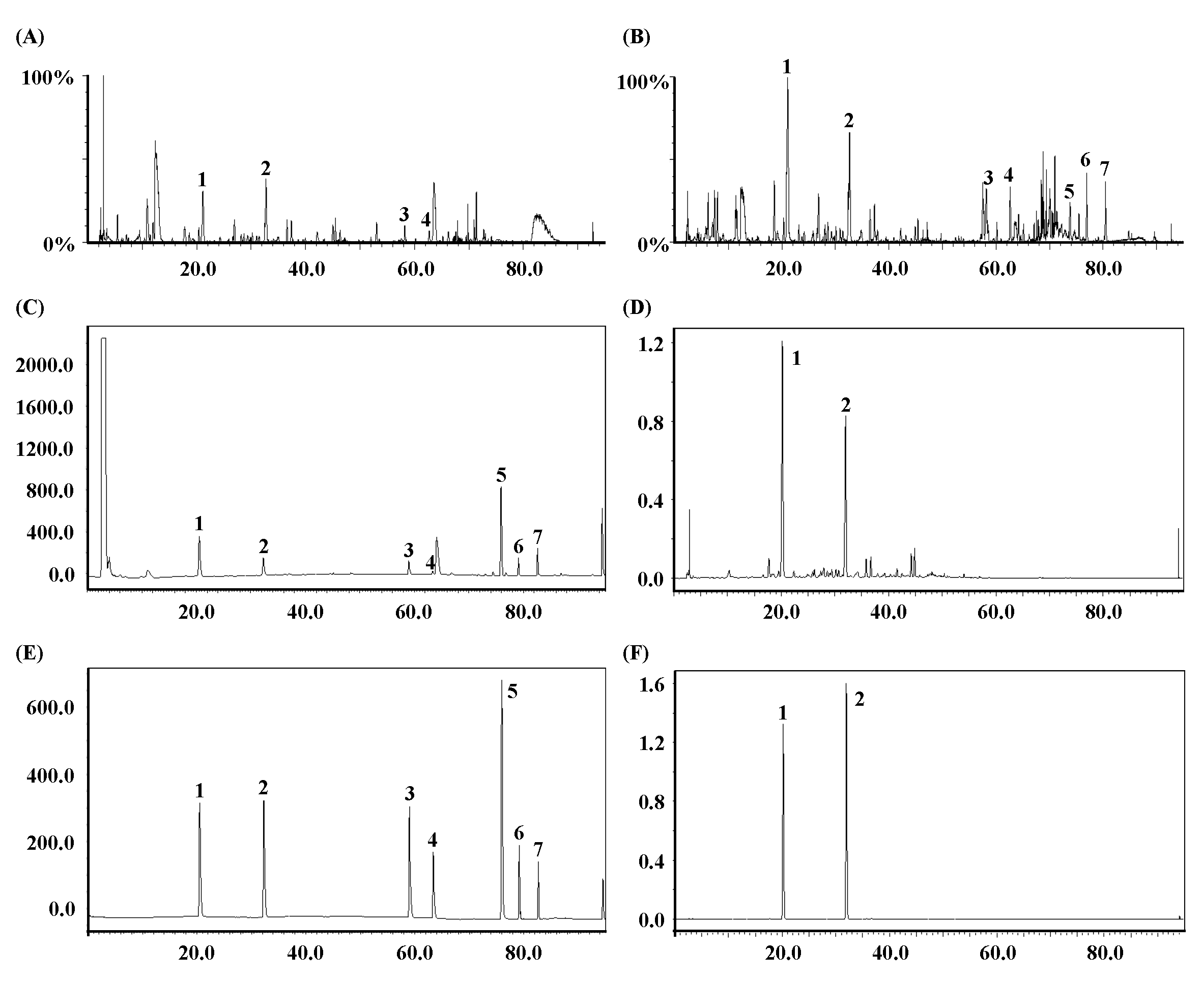

Supplement: Supplementary file 2 [file Image1.TIFF]

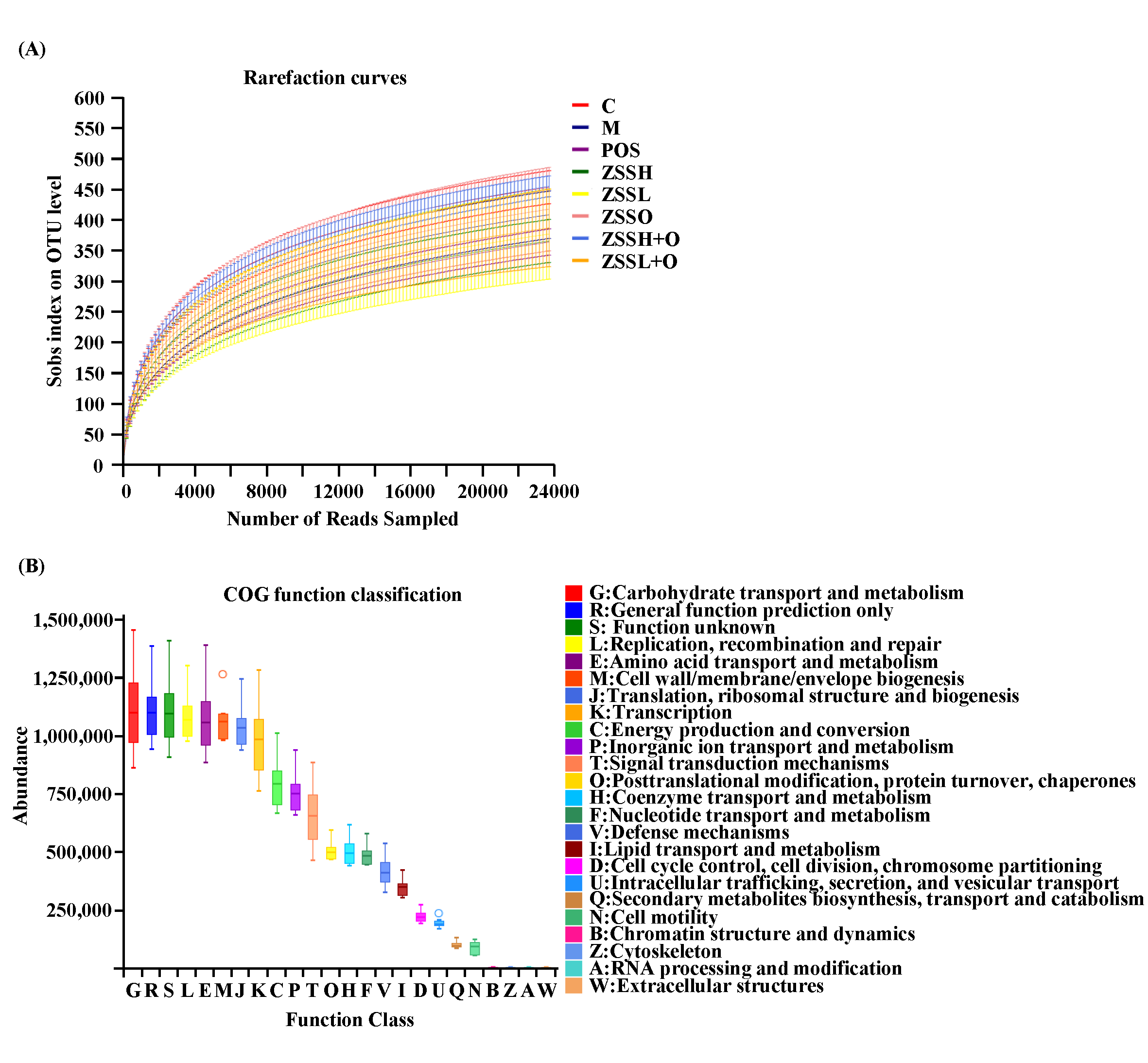

Supplement: Supplementary file 3 [file Image2.TIFF]
