# Supplementary material for: Ziziphus jujuba Mill. var. spinosa (Bunge) Hu ex H. F. Chou Seed Ameliorates Insomnia in Rats by Regulating Metabolomics and Intestinal Flora Composition
Source: Front Pharmacol. 2021 Jun 16;12:653767. doi: 10.3389/fphar.2021.653767 (PMC8241942; doi:10.3389/fphar.2021.653767)
Supplement: Supplementary file 4 [file DataSheet1.docx]

Supplementary Material

# Supplementary Data

**Method S1** Qualitative and quantitative analysis of ZSS extract

The qualitative and quantitative analysis of the composition of ZSS extract was performed on UPLC-Q-TOF/MS (Waters, USA) and HPLC-PDA-ELSD (Waters, USA; Alltech, USA), respectively. In detail, chromatographic separations were performed on a XBridge ® C18 column (4.6 × 150 mm, 3.5 μm, Waters, USA) using solvent A (0.2% formic acid aqueous solution) and B (acetonitrile) as the mobile phase for gradient elution. The flow rate was 0.6 mL/min, column temperature was 30 °C and injection volume was 10 μL. The gradient elution was carried out at 0−42 min, 90−77% A; 42−44 min, 77−72% A; 44−64 min, 72−60% A; 64−66 min, 60−20% A; 66−85 min, 20−0% A; 85−90 min, 0% A; 90−91 min, 0−90% A; 91−95 min, 90% A.

The PDA detection wavelength range of UPLC and HPLC was set at 190−400 nm. The Q-TOF mass spectrometer was operated with both positive and negative ionization modes by scanning over the m/z range 100−1,500Da. The conditions were as follows: capillary voltage, 4.5 kV; collision energy, 20−40 eV; desolvation temperature, 400 °C; ion source temperature, 150 °C; desolvation/cone gas flow of 800 L/h, 50 L/h; scan time, 0.3 s; and interscan time, 0.014 s. High purity nitrogen and leucine-enkephalin (ESI+: 556.2289 m/z, ESI−: 554.2205 m/z) were gas collision and locked mass solution, respectively. The parameters of the ELSD detector were as follows: the drift tube temperature was 80 °C, the gas flow rate was 2.7 mL/min, and gain value was set to 8.

**Method S2** Short-chain fatty acid analysis of fresh feces and cecum contents

The short-chain fatty acid content determination of fresh feces and cecum contents was performed on GC-FID (Clarus® 680, PerkinElmer, USA). In detail, chromatographic separations were performed on a HP-5MS capillary column (30 m × 0.250 mm, 0.25 μm, Agilent Technologies, Inc., USA). The injection volume was 2 μL, and the conditions were as follows: channel sampling rate, 12.5 min/s; carrier gas, helium; split ratio, 10:1; air flow rate, 400 ml/min; H2 flow rate,40 ml/min. The injector temperature was 250 °C, FID detector temperature was 250 °C, and the oven ramp was programmed: the initial temperature was 100 °C, holding for 2.5 min, then heating to 230 °C at a rate of 50 °C/min and holding for 2 minutes.

# Supplementary Figures and Tables

**Table S1** Identiﬁcation of chemical constituents of ZSS extract

| **No.** | **t_R_ (min)** | **λ_max_ (nm)** | **Selected ion** | **MS fragments (m/z)** | **Identified components** |
| --- | --- | --- | --- | --- | --- |
| 1 | 21.13 | 336; 270 | [M+H] ^+^  609.1822 | 489.1396, 447.1291, 429.1188, 381.0977, **327.0880**, 297.0771, 267.0669 | spinosin |
|  |  |  | [M–H] ^–^  607.1669 | 487.1233, 427.1029, 292.0411, 240.9046 |  |
| 2 | 32.72 | 330; 272 | [M+H] ^+^  785.2278 | 755.2156, 665.1863, 494.1985, 393.0980, **327.0877**, 297.0775 | 6′′′-feruloyl spinosin |
|  |  |  | [M–H] ^–^  783.2114 | 753.1989, 607.1645, 525.2096, 427.1082, 292.0370, 210.9435 |  |
| 3 | 58.20 | / | [M+H] ^+^  1207.6140 | 1061.5553, 909.7021, 733.4527, 587.3943, 565.3883, **455.3529**, 437.3411, 369.2787, 315.2308, 159.1177 | jujuboside A |
|  |  |  | [M–H] ^–^  1205.5891 | 1119.706, 576.2993, 479.3345, 381.9259, 329.2350, 211.9031 |  |
| 4 | 62.70 | / | [M+H] ^+^  1045.5585 | 895.5042, 749.4449, 733.4509, 697.4300, 587.3953, 565.3886, **455.3529**, 437.3418, 369.2827, 315.2307, 123.1192 | jujuboside B |
|  |  |  | [M–H] ^–^  1043.5375 | 957.5003, 795.4534, 662.5888, 595.4328, 370.5114, 330.2436, 171.1039 |  |
| 5 | 73.85 | / | [M–H] ^–^  455.3526 | 409.2376, 325.1859, 134.8952 | betulinic acid |
| 6 | 76.93 | / | [M–H] ^–^  279.2332 | 116.9302 | linoleic acid |
| 7 | 80.46 | / | [M–H] ^–^  281.2492 | 199.8525 | oleic acid |

**Table S2** Investigation of the ZSS extract linear relationship

| **Analytes** | **Regression equation** | **r^2^** | **Linear range (μg/mL)** |
| --- | --- | --- | --- |
| spinosin | y=33392x+163580 | 0.9988 | 17.5-1120 |
| 6′′′-feruloylspinosin | y=35923x+154514 | 0.9985 | 17.35-1110 |
| jujuboside A | y=1.4624x+2.6131 | 0.9975 | 17.5-1120 |
| jujuboside B | y=1.4461x+2.6488 | 0.9989 | 15.63-1000 |
| betulinic acid | y=1.5372x+2.7512 | 0.9976 | 16.41-1050 |
| linoleic acid | y=1.8242x+1.5816 | 0.9927 | 9.57-612.5 |
| oleic acid | y=1.4841x+2.5826 | 0.9974 | 10.23-655 |

**Table S3** Precision, stability, repeatability and recovery (n=6) of ZSS extract

| **Analytes** | **Precision (RSD/%)** | | **Stability (RSD/%, n=6)** | **Repeatability**  **(RSD/%, n=6)** | **Recovery (%, n=6)** | |
| --- | --- | --- | --- | --- | --- | --- |
|  | **Intraday (n=6)** | **Interday (n=6)** |  |  | **Mean** | **RSD** |
| spinosin | 2.94 | 3.58 | 1.00 | 0.38 | 96.87 | 0.35 |
| 6′′′-feruloylspinosin | 2.81 | 3.43 | 0.67 | 0.26 | 96.14 | 0.72 |
| jujuboside A | 2.91 | 3.43 | 2.20 | 0.58 | 94.46 | 1.03 |
| jujuboside B | 4.42 | 6.15 | 2.27 | 0.58 | 95.54 | 1.41 |
| betulinic acid | 4.89 | 5.09 | 2.21 | 1.23 | 97.36 | 0.93 |
| linoleic acid | 4.41 | 3.90 | 1.91 | 0.94 | 94.31 | 1.60 |
| oleic acid | 4.80 | 5.09 | 1.69 | 1.54 | 94.03 | 1.13 |

**Table S4** Potential biomarkers selected and identified between model and control rats

| **No. ^a^** | **t_R_ (min)** | **Mass (m/z)** | **Metabolite** | **Trend ^b^** | **Ion mode** | **HMDB** |
| --- | --- | --- | --- | --- | --- | --- |
| PM1 | 1.81 | 154.0417 | 3-Sulfinoalanine | ↑ | + | HMDB0000996 |
| PM2 | 10.36 | 782.5727 | PE (22:2(13Z,16Z)  /P-18:1(11Z)) | ↓ | + | HMDB0009578 |
| PM3 | 4.33 | 463.2143 | 6-Dehydrotestosterone glucuronide | ↑ | + | HMDB0010337 |
| PM4 | 4.33 | 465.2169 | 15-Hydroxynorandrostene-3,17-dione glucuronide | ↑ | + | HMDB0010353 |
| PM5 | 10.74 | 305.2461 | Arachidonic acid | ↑ | + | HMDB0001043 |
|  |  | 303.2327 |  |  | - |  |
| PM6 | 11.97 | 160.8417 | Phosphoroselenoic acid | ↑ | - | HMDB0003840 |
| PM7 | 4.48 | 437.2896 | LysoPA (0:0/18:0) | ↑ | - | HMDB0007850 |
| UM1 | 1.72 | 112.0762 | Histamine | ↑ | + | HMDB0000870 |
| UM2 | 2.23 | 126.0224 | Taurine | ↓ | + | HMDB0000251 |
| UM3 | 5.95 | 133.0962 | Ornithine | ↑ | + | HMDB0000214 |
| UM4 | 4.64 | 148.0412 | L-Glutamic acid | ↓ | + | HMDB0000148 |
| UM5 | 4.18 | 147.0936 | L-Glutamine | ↓ | + | HMDB0000641 |
| UM6 | 7.88 | 149.0969 | Mevalonic acid | ↑ | + | HMDB0000227 |
| UM7 | 2.86 | 154.0424 | Dopamine | ↑ | + | HMDB0000073 |
| UM8 | 2.44 | 162.0529 | Aminoadipic acid | ↑ | + | HMDB0000510 |
| UM9 | 2.22 | 167.1038 | 1-Methylxanthine | ↓ | + | HMDB0010738 |
| UM10 | 12.65 | 456.1706 | 5,10-Methenyltetra-  hydrofolic acid | ↓ | + | HMDB0001354 |
| UM11 | 0.88 | 151.0633 | D-Xylulose | ↓ | + | HMDB0001644 |
| UM12 | 2.75 | 149.0601 | D-Xylose | ↓ | - | HMDB0001644 |
| UM13 | 6.27 | 289.1119 | Argininosuccinic acid | ↑ | - | HMDB0000052 |
| UM14 | 2.57 | 119.0497 | Phenylacetaldehyde | ↑ | - | HMDB0006236 |
| UM15 | 1.86 | 137.0233 | Urocanic acid | ↓ | - | HMDB0000301 |
| UM16 | 2.56 | 163.0388 | Phenylpyruvic acid | ↑ | - | HMDB0000205 |
| UM17 | 3.81 | 371.1338 | Biocytin | ↑ | - | HMDB0003134 |

a: PM: metabolites from plasma; UM: metabolites from urine.

b: The trend is model group vs control group: ↑, increase; ↓, decrease.

**Table S5** Prediction of metabolic pathways from samples

| **No.** | **KEGG pathway** | **KEGG pathway**  **(upper level)** | **Abundance value** |
| --- | --- | --- | --- |
| 1 | Carbohydrate Metabolism | Metabolism | 1456123 |
| 2 | Amino Acid Metabolism | Metabolism | 1372010 |
| 3 | Energy Metabolism | Metabolism | 883245 |
| 4 | Metabolism of Cofactors and Vitamins | Metabolism | 638681 |
| 5 | Nucleotide Metabolism | Metabolism | 636354 |
| 6 | Lipid Metabolism | Metabolism | 376536 |
| 7 | Glycan Biosynthesis and Metabolism | Metabolism | 369048 |
| 8 | Enzyme Families | Metabolism | 330927 |
| 9 | Metabolism of Terpenoids and Polyketides | Metabolism | 250549 |
| 10 | Xenobiotics Biodegradation and Metabolism | Metabolism | 221328 |
| 11 | Metabolism of Other Amino Acids | Metabolism | 212201 |
| 12 | Membrane Transport | Environmental Information Processing | 1523252 |
| 13 | Replication and Repair | Genetic Information Processing | 1385355 |
| 14 | Translation | Genetic Information Processing | 901526 |
| 15 | Transcription | Genetic Information Processing | 382209 |
| 16 | Folding, Sorting and Degradation | Genetic Information Processing | 367780 |
| 17 | Poorly Characterized | Unclassified | 694417 |
| 18 | Cellular Processes and Signaling | Unclassified | 557143 |
| 19 | Genetic Information Processing | Unclassified | 383305 |
| 20 | Metabolism | Unclassified | 338214 |

**Table S6** Investigation of SCFA linear relationship

| **Analytes** | **Regression equation** | **r^2^** | **Linear range (mg/mL)** |
| --- | --- | --- | --- |
| acetic acid | y=33306x-13799 | 0.9996 | 0.161-20.67 |
| propionic acid | y=50433x-21898 | 0.9993 | 0.270-34.62 |
| isobutyric acid | y=48290x-7835.9 | 0.9998 | 0.149-19.05 |
| butyric acid | y=55748x-15519 | 0.9995 | 0.199-25.50 |
| isovaleric acid | y=64828x-17592 | 0.9995 | 0.205-26.24 |
| valeric acid | y=56233x-28048 | 0.9992 | 0.223-28.60 |

**Table S7** Stability, repeatability, precision and recovery (n=6) of SCFA

| **Analytes** | **Precision (RSD/%)** | | **Stability (RSD/%, n=6)** | **Repeatability**  **(RSD/%, n=6)** | **Recovery**  **(%, n=6)** | |
| --- | --- | --- | --- | --- | --- | --- |
|  | **Intraday (n=6)** | **Interday (n=6)** |  |  | **Mean** | **RSD** |
| acetic acid | 3.76 | 4.55 | 5.77 | 3.09 | 88.46 | 6.41 |
| propionic acid | 3.89 | 4.43 | 6.06 | 4.93 | 93.73 | 4.46 |
| isobutyric acid | 3.77 | 4.33 | 7.89 | 5.43 | 85.19 | 3.22 |
| butyric acid | 2.93 | 3.01 | 3.08 | 4.66 | 88.01 | 4.09 |
| isovaleric acid | 2.90 | 3.61 | 2.24 | 3.54 | 105.17 | 7.60 |
| valeric acid | 3.69 | 3.78 | 5.07 | 5.02 | 85.95 | 4.29 |

**
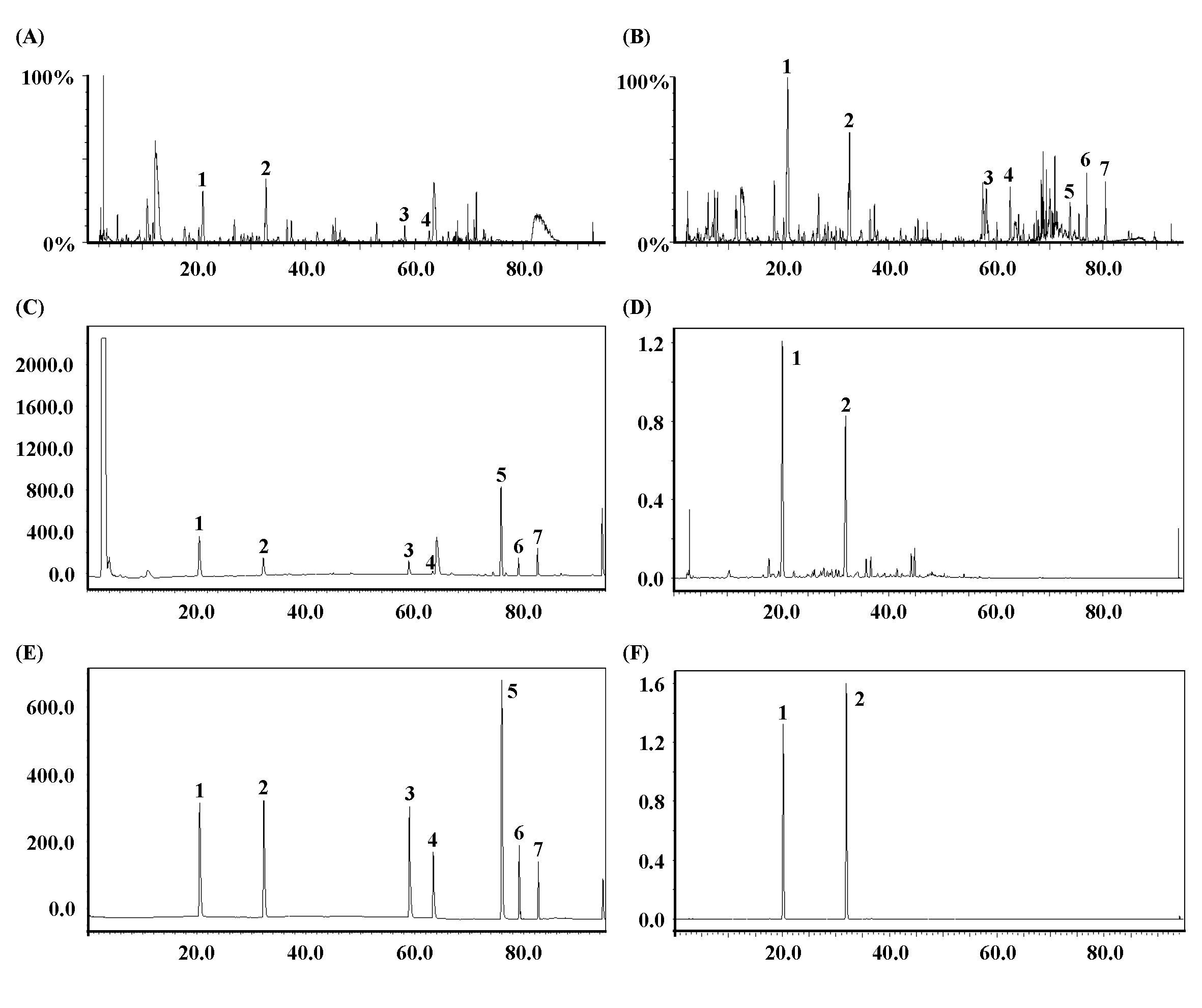
**

**Figure S1** The UPLC-Q-TOF/MS total ion chromatogram in positive (A) and negative (B) ion mode and HPLC-ELSD (C, E)-PDA (D, F) chromatogram of ZSS extract (C, D), and spinosin (1), 6′′′-feruloylspinosin (2), jujuboside A (3), jujuboside B (4), betulinic acid (5), linoleic acid (6) and oleic acid (7) references (E, F)


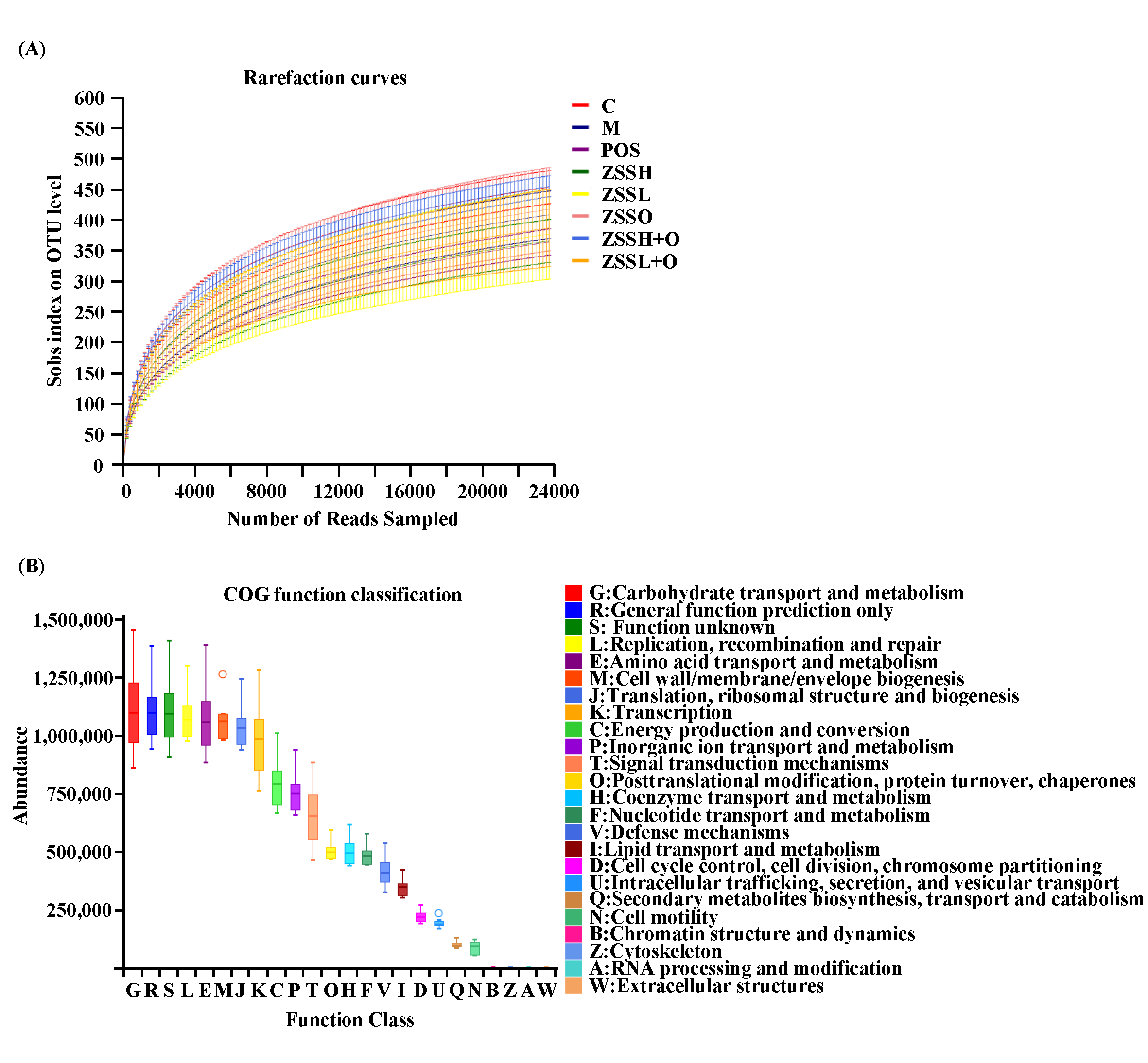


**Figure S2** Rarefaction curve of sobs index of each group (A) and metabolic function prediction of community samples (B)


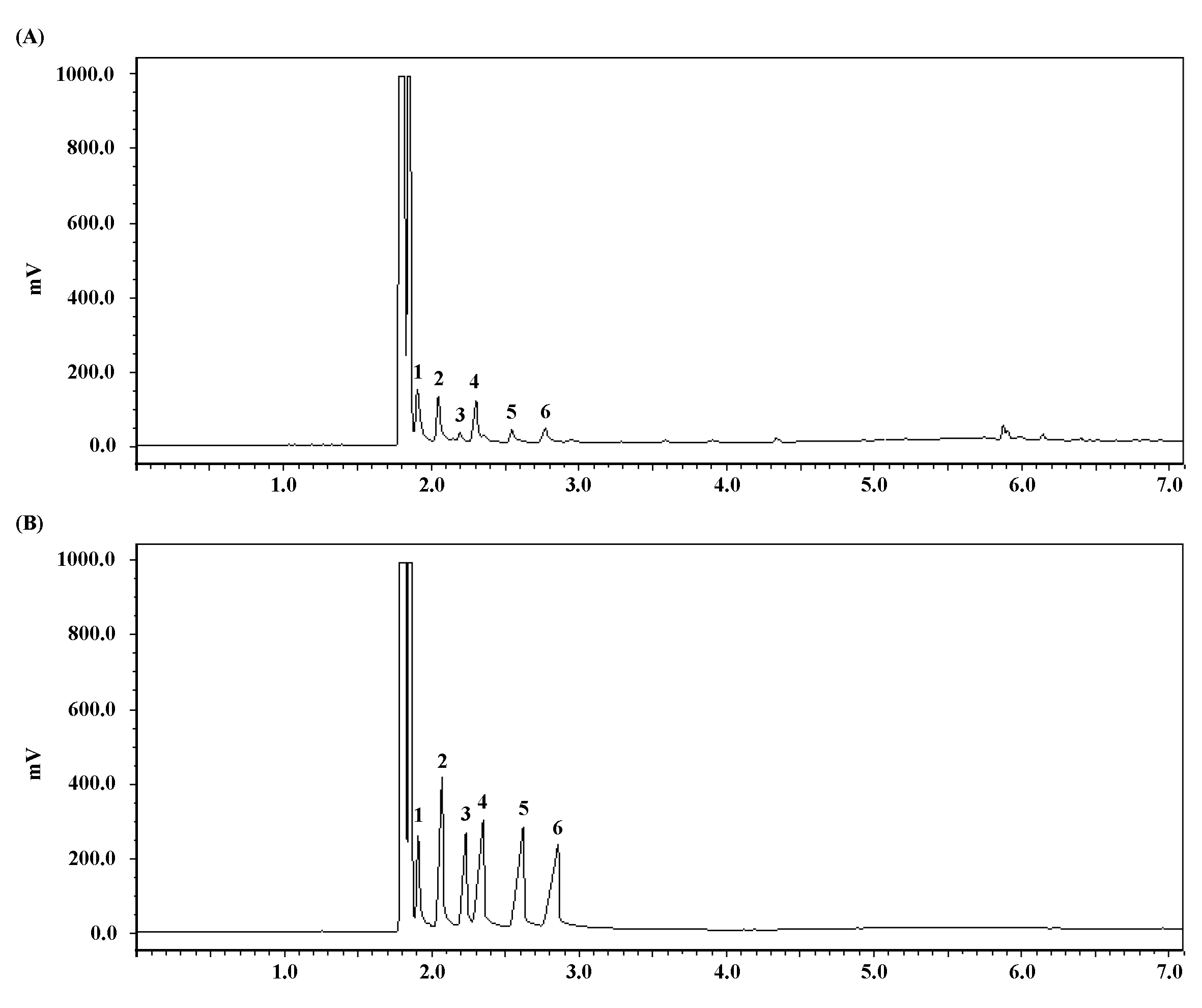


**Figure S3** GC-FID chromatogram of short-chain fatty acids in feces (A), and acetic acid (1), propionic acid (2), isobutyric acid (3), butyric acid (4), isovaleric acid (5) and valeric acid (6) references (B)
